# Genomic Analysis of *Shewanella* sp. O23S—The Natural Host of the pSheB Plasmid Carrying Genes for Arsenic Resistance and Dissimilatory Reduction

**DOI:** 10.3390/ijms20051018

**Published:** 2019-02-26

**Authors:** Witold Uhrynowski, Monika Radlinska, Lukasz Drewniak

**Affiliations:** 1 Laboratory of Environmental Pollution Analysis, Faculty of Biology, University of Warsaw, Miecznikowa 1, 02-096 Warsaw, Poland; w.uhrynowski@biol.uw.edu.pl; 2 Department of Virology, Institute of Microbiology, Faculty of Biology, University of Warsaw, Miecznikowa 1, 02-096 Warsaw, Poland; m.radlinska@biol.uw.edu.pl

**Keywords:** *Shewanella* spp., arsenic, dissimilatory arsenate reduction, heavy metals resistance, genome, mobilome, plasmid pSheB, phage

## Abstract

*Shewanella* sp. O23S is a dissimilatory arsenate reducing bacterial strain involved in arsenic transformations within the abandoned gold mine in Zloty Stok (SW Poland). Previous physiological studies revealed that O23S may not only release arsenic from minerals, but also facilitate its immobilization through co-precipitation with reduced sulfur species. Given these uncommon, complementary characteristics and the application potential of the strain in arsenic-removal technologies, its genome (~5.3 Mbp), consisting of a single chromosome, two large plasmids (pSheA and pSheB) and three small plasmid-like phages (pSheC-E) was sequenced and annotated. Genes encoding putative proteins involved in heavy metal transformations, antibiotic resistance and other phenotypic traits were identified. An in-depth comparative analysis of arsenic respiration (*arr*) and resistance (*ars*) genes and their genetic context was also performed, revealing that pSheB carries the only copy of the *arr* genes, and a complete *ars* operon. The plasmid pSheB is therefore a unique natural vector of these genes, providing the host cells arsenic respiration and resistance abilities. The functionality of the identified genes was determined based on the results of the previous and additional physiological studies, including: the assessment of heavy metal and antibiotic resistance under various conditions, adhesion-biofilm formation assay and Biolog^TM^ metabolic preferences test. This combined genetic and physiological approach shed a new light on the capabilities of O23S and their molecular basis, and helped to confirm the biosafety of the strain in relation to its application in bioremediation technologies.

## 1. Introduction

Extreme environments, including heavy metal polluted mining sites, constitute important research areas plentiful in species with interesting adaptive traits [1]. The ancient gold and arsenic mine in Zloty Stok, located in southwestern Poland, has already been described as a source of arsenic resistant microorganisms, capable of both oxidation of As(III), e.g., *Ensifer* (*Sinorhizobium*) sp. M14 [2,3], and dissimilatory reduction of As(V), e.g., *Aeromonas* sp. O23A [4]. Due to the differences in the solubility between arsenites and arsenates, the latter being less soluble in water, arsenic oxidizing bacteria are regarded as beneficial in arsenic-contaminated water treatment technologies, especially when they are applied in combination with physical and chemical methods, as in the MicroAsOx technology [3]. In turn, dissimilatory arsenate reducing bacteria (DARB) are considered the main arsenic mobilizers in the environment [5,6], and their presence usually contributes to increasing of the concentration of arsenic in waters, and especially underground waters, such as mine effluents [7,8,9]. However, this general tendency may not always be accurate. Previous studies have indicated that some DARB species, including the two strains isolated from the Zloty Stok mine: *Aeromonas* sp. O23A and *Shewanella* sp. O23S, may contribute not only to mobilization, but also immobilization of arsenic [4,10]. Thus, DARB activity should not always be considered an environmental hazard. In fact, DARB may find application in bioremediation, as they can facilitate controlled removal of arsenic from solid material, including minerals, waste residues [11], and contaminated soil [12]. This capability of mobilization of heavy metals by DARB can be used to selectively remove the toxic species by applying adequate red-ox potential, for example in redox-stat reactors [13].

The actual potential of bacterial strains may be assessed by basic physiological research or, to obtain a more complete view, by genomic investigation, followed by functional analyses. Genomic analyses allow to determine the gene pool of a (micro)organism, including the genes coding for both the beneficial characteristics, such as tolerance to extreme conditions (e.g., low temperature, or highly acidic or alkaline pH) and potentially undesirable traits, including pathogenicity and antibiotic resistance [3,14]. Such traits may not always be identified by physiological analyses alone, especially as the expression of some genes may occur only under particular physico-chemical conditions [14]. Moreover, analyses performed on sequencing data may also help to identify the potential horizontal gene transfer (HGT) events which played a role in the shaping of the genome, as well as to determine the mobility of genes or gene modules, what may be crucial in the biosafety assessment of strains showing particular application potential [15].

Apart from biosafety, applicability of (micro)organisms in bioremediation and water treatment technologies is greatly dependent on the survivability in a polluted niche. In the case of remediation of heavy metal contaminated areas using bacterial strains, it is important to analyze the presence and functionality of heavy metal resistance (*hmr*) genes, encoding, i.a., efflux pumps and transporters, as well as other genes which protein products are involved in metal transformations and metabolism. In the case of arsenic resistance, a functional set of *ars* genes is required, usually in the form of *ars(R)DABC* operon consisting of an *arsC* gene encoding reductase, *arsA-B* encoding efflux pump components, and *arsD*/*arsR* coding for operon regulators [16,17]. Arsenic resistance genes were found both on chromosomes and extrachromosomal replicons of bacteria, e.g., plasmid R46 [18]. In turn, respiratory arsenate reduction is conditioned upon the expression of *arrA* and *arrB* genes, encoding a periplasmic dissimilatory arsenate reductase and an iron-sulfur protein involved in electron transport, respectively [19,20]. Although the Arr complex has been extensively studied for many years, only recently its crystallographic structure has been solved [21].

The availability of molecular analysis tools facilitates identification of yet novel *arrA* gene phylotypes [22,23], indicating high variability of the arsenic respiration genes. However, complete genomes of only a few arsenate reducers, for which this ability has been experimentally confirmed, are available in the databases, including bacteria: *Herminiimonas arsenicoxydans*, and *Aeromonas* sp. O23A [14,24] as well as dissimilatory arsenate reducing archaeon *Pyrobaculum arsenaticum* [25].

For the object of this study, *Shewanella* sp. O23S, a series of physiological analyses has already been carried out, indicating optimum growth conditions, general heavy metals resistance capabilities and assessment of the application potential of the strain [10]. It was also found that O23S is the host of a natural vector of arsenic resistance (*ars*) and respiration (*arr*) genes. This plasmid, named pSheB, can be potentially transferred to other strains and provide them arsenic resistance and metabolism abilities (US Patent no. 9328397B2). However, thorough analysis of the sequence of this plasmid, as well as the chromosome and other replicons of O23S has not been performed yet. Therefore, the aim of this study is to provide a complex analysis of the genome of O23S, with special focus on genes potentially related to heavy metal resistance and metabolism. The arsenic resistance and respiration modules were compared with analogous genes found in other *Shewanella* spp. and DARB strains, including *Aeromonas* sp. O23A isolated from the same environment as O23S. Particular attention was also given to the mobilome of O23S, and especially, the plasmid pSheB—being the natural vector of *ars* and *arr* genes. Additional physiological tests, including adhesion-biofilm formation assay as well as antibiotic resistance and in depth Biolog^TM^ analyses have been performed. This study therefore is complementary to the previous work on O23S; it provides novel information on the traits encoded within the O23S genome and confirms the functionality of the indicated genes. The obtained results further explain the role of *Shewanella* sp. O23S in the arsenic cycle in the environment and its impact on the microbial community inhabiting the Zloty Stok mine. They are also crucial for the determination of the actual application potential and biosafety of the strain.

## 2. Results and Discussion

### 2.1. General Features of the Shewanella sp. O23S Genome

The genome of *Shewanella* sp. O23S was sequenced using two independent approaches and platforms: PacBio and Illumina. The obtained reads were manually curated, and the genome sequencing and assembly completeness and correctness were additionally verified by using the pNGS FOS fosmid library of the O23S genome created in *Escherichia coli* F96401-1.

**Figure 1:**
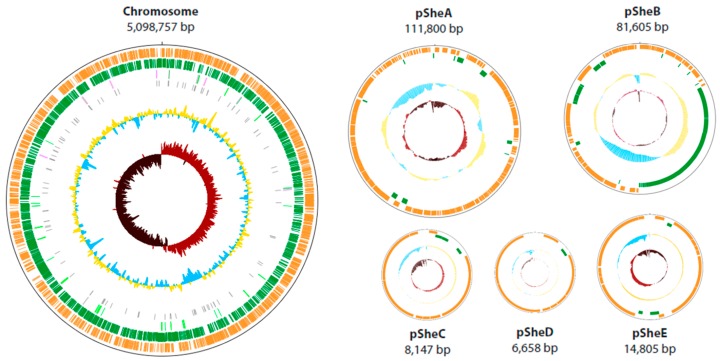
Physical maps of the chromosome and plasmids of *Shewanella* sp. O23S. Open reading frames are indicated by orange and dark green blocks. The innermost circle represents GC skew, whereas the larger ones—GC content, repeat regions and RNA genes, respectively.

It was found that the genome of O23S, of the total size of 5,321,772 bp is composed of a single circular chromosome (5,098,757 bp), two large circular plasmids: pSheA (111,800 bp) and pSheB (81,605 bp), and 3 smaller plasmid-like phages: pSheC (8147 bp) and pSheD (6658 bp), which together co-integrate to form another replicon, pSheE (14,805 bp). Physical map and general features of the genome are presented below (Figure 1, Table 1). The visualization of the plasmids isolated from O23S cells is shown in Figure S1.

Automatic annotation using RAST on the PATRIC 3.4.13 platform [26] identified 4948 genes (4654 within the chromosome and 157, 91, 12, 11, and 23 in the plasmids pSheA-E, respectively) with an average length of 920 bp. The smallest distinguished gene (90 bp), encoding a hypothetical protein identical to a putative protein of *Shewanella putrefaciens* (acc. no. RBP80366.1), and the largest one (12,804 bp), identical in 93% with an adhesin of *Shewanella baltica* OS185 (acc. no. WP_041411340.1), were both found within the *Shewanella* sp. O23S chromosome. Additionally, 106 tRNA genes and 10 clusters of 16S-23S-5S rRNA (plus one additional 5S rRNA-encoding gene, O23S_r00022c, located between 4,563,479–4,563,600) were identified within the O23S chromosome (Table 1). No rRNA- or tRNA-encoding genes were found on the plasmids. The chromosome annotation allowed to distinguish 3421 genes encoding putative proteins, of which 1003 were assigned with EC numbers and 709 were mapped to KEGG pathways. The remaining 1233 genes found within the chromosome encode hypothetical proteins. The analysis revealed that 4154 putative proteins encoded within the chromosome belong to the genus-specific protein families and 4220 proteins belong to the cross-genus protein families.

**Figure 2:**
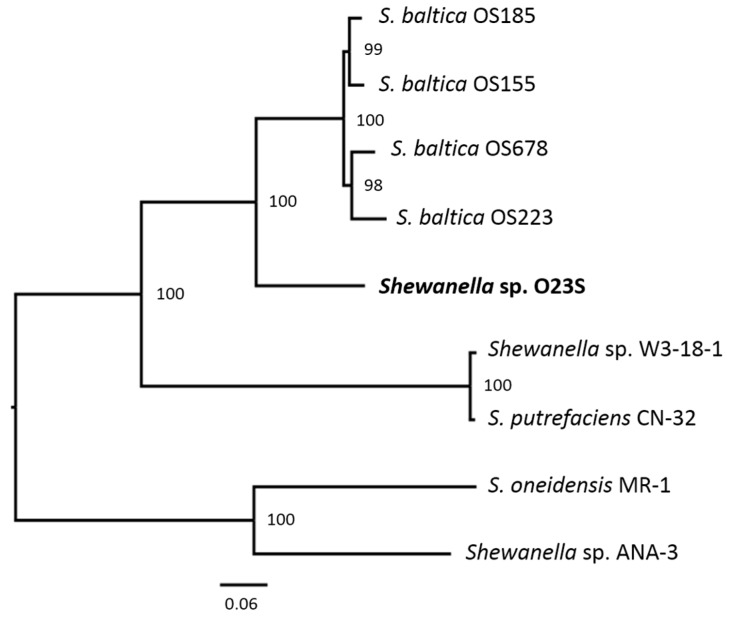
Phylogenetic tree of *Shewanella* spp. to which *Shewanella* sp. O23S shows the highest resemblance. The phylogenetic placement of O23S was determined based on the comparison of global protein families selected from the representative closest genomes identified as a part of PATRIC automatic annotation. The bootstrap support values are shown next to the branches.

Based on the phylogenetic analyses performed for the set of proteins classified to the cross-genus (global) families, the closest reference and representative genomes to that of *Shewanella* sp. O23S were found to belong to *Shewanella baltica* strains OS185, OS155, OS223, and OS678, all of which have been isolated between 1986 and 1998 from the same marine environment (the Baltic Sea), and later sequenced by Caro-Quintero et al. [27]. The genomes of these strains range from 5.0 to 5.4 Mb in size, with the GC content (G+C) of approx. 45–46%. The genome of O23S also fits within these limits. Interestingly, the Baltic Sea isolates have been described as remarkably prone to re-shaping of the genomes in HGT events [28], and they differ in the number of autonomous replicons found within their cells (from 1 to 4). The genomic composition of O23S strain most resembles that of *S. baltica* OS155, which also carries a ~5.1 Mbp chromosome, 2 large plasmids (116.8 and 74 kbp), and 2 smaller plasmids (16.8 and 8.0 kpb). Apart from the *S. baltica* strains, phylogenetic analysis also revealed a close resemblance of the O23S genome to that of *S. oneidensis* MR-1, *Shewanella* sp. W3-18-1, *S. putrefaciens* CN-32 and *Shewanella* sp. ANA-3. The phylogenetic tree indicating the relative distances between the strains is shown in Figure 2.

### 2.2. Mobilome of O23S

Isolation of plasmid DNA by cesium chloride centrifugation and electrophoretic separation of the obtained DNA allowed to identify 2 large plasmids present in *Shewanella* sp. O23S cells pSheA and pSheB, as well as 3 smaller phage-like replicons, named pSheC, pSheD, and pSheE (Figure S1). After obtaining the nucleotide sequence of the replicons, a detailed bioinformatic analysis was carried out with particular attention to the replicative, partition, stabilizing, mobilization/conjugal transfer modules constituting the backbone of the plasmids, as well as the potential modules determining the phenotypic traits, and, especially, heavy metal resistance and metabolism genes.

#### 2.2.1. Plasmid pSheA

The largest of the extrachromosomal replicons of *Shewanella* sp. O23S, named pSheA, has a size of 111.8 kb, and comprises 157 open reading frames (ORFs) (Table 1). Among the indicated genes only about 30% encode proteins for which probable function could be determined based on comparative analyses. The majority of these ORFs code for putative proteins, which may play a role in (i) replication (i.a., ORFs 1, 3, 10, and 105), (ii) conjugational transfer (ORFs 107–127), (iii) and stable maintenance of the plasmid in bacterial population, including the partitioning (ORFs 90–91 and 99–100) and multimer resolution (ORF 133) systems components. Together, these ORFs constitute the backbone structure of the pSheA plasmid discussed in detail below. The complete list of ORFs identified within the plasmid pSheA, with the indication of their potential function is presented in Supplementary Materials (Table S1).

The replication system of pSheA is complex, as it comprises two Rep proteins, RepA and RepB, encoded by ORFs 1 and 3, respectively. The former protein shares highest homology with a hypothetical protein of *Vibrio parahaemolyticus*, but is also similar (in 54%) to the RepA protein of *Providencia rettgeri* (acc. no ARV76042.1). The latter protein is in 89% identical with the initiator RepB protein of the pSBAL11702 plasmid of *Shewanella baltica* OS117 (acc. no. AEH16402.1). Analyses also indicated the presence of genes coding for a potential helicase containing a DEAD/DEAH box domain (ORF 10, in 91% identical with that encoded by *Shewanella* sp. POL2; acc. no. WP_050991317.1), as well as a putative DNA topoisomerase III (ORF 105, in 35% identical with that of *Pseudomonas psychrotolerans*; acc. no. KTT64086.1). Both these proteins may also play a role in pSheA replication: its initiation by facilitating the separation of DNA and the removal of DNA supercoils formed during replication, respectively. Moreover, it was found that pSheA encodes two copies of antirestriction protein ArdR (ORF 52 and ORF 61), in ~80% identical with that of *S. putrefaciens*. These putative protein products may play a role in overcoming the host restriction barrier in plasmids, as it was shown for other proteins belonging to the same family [29].

The conjugational transfer module of the plasmid pSheA (coordinates 74,683–94,245) is comprised of genes coding for several putative proteins, e.g., TraD (ORF 112), TraG (ORF 110), TraP (ORF 107), and several integrating conjugative elements (e.g., ORFs 126–127). In addition, a number of genes coding for hypothetical proteins are also assumed to be a part of the conjugational transfer module due to their co-localization with genes encoding more defined Tra homologs and/or the presence of a common domain TIGR03751 in the putative protein product. However, the limited similarity of the putative pSheA protein products to the known conjugational transfer module components may indicate that the system is impaired. Therefore, the functionality of the module should be confirmed by *in vitro* studies.

Apart from the replication and conjugative transfer module two partitioning systems have been identified in the pSheA plasmid (ORFs 90–92 and ORFs 99–100). Both these systems encode putative ParA–B proteins showing ~70% similarity to the system encoded by *S. putrefaciens* and ~50% similarity to that of *Vibrio cholerae*, respectively. Interestingly, the product of ORF 79 also shows 71% similarity to the ParA family protein of *Salmonella enterica*. In turn, the multimer resolution system encoded by the plasmid pSheA comprises a putative resolvase, showing similarity (78%) to the serine resolvase of *Acinetobacter baumanii* (acc. no. SYY08110.1). In addition, genes encoding putative components of a restriction-modification system, including a DNA cytosine methyltransferase (ORF 89) and methyltransferase type 11 (ORF 137) were found on pSheA.

The plasmid pSheA also comprises a set of genes (ORFs 20, 22), which putative protein products contain conserved PulE/PulG domains, indicating their similarity to the type II secretion system proteins, often associated with pathogenicity [30]. Although pSheA carries only selected proteins of the system which is likely non-functional, complete systems of this type have been identified in *Shewanella* spp. representatives, e.g., in fish pathogen *S. putrefaciens* [31].

Among the genes encoding other phenotypic traits are ORF 68, which product shows 55% similarity to putative FMN-dependent luciferase-like monooxygenase of *Enterobacter* spp. (acc. no. KLP52955.1), ORF 76—encoding a hypothetical protein with limited similarity to CPBP family intramembrane metalloprotease (*Clostridium frigidicarnis;* acc. no. SFB30445.1) and others. The remaining genes identified in the pSheA (~100 ORFs) encode hypothetical proteins or proteins which function is unknown and could not be defined based on comparative analyses or co-localization with other structural modules. Several of them, however, contain transmembrane domains and signal sequences, possibly indicating their location within the cell membrane and/or periplasm.

Interestingly, the results of comparative analyses indicate that pSheA has a mosaic-like structure. For example, this phenomenon may be observed for the replication and partitioning systems, which are both present in two copies, each resembling a module found in a different host, belonging either to *Shewanella* spp. and *Vibrio* spp. In fact, approx. 30% of all the identified gene products of pSheA show highest similarity to those encoded in the genomes of *Vibrio* spp., and especially *Vibrio parahaemolyticus*. These are interspersed with the genes encoding proteins identified in other *Shewanella* spp., mainly *Shewanella xiamenensis*, *S. baltica* and *Shewanella* sp. POL2. Such a plasmid structure may indicate the occurrence of HGT events between strains belonging to these genera.

#### 2.2.2. Plasmid pSheB

Although pSheA is the largest of the O23S plasmids, it is the second large plasmid—pSheB (81 kbp) which carries more genes coding for proteins which probable function could be determined by comparative analyses. In general, the plasmid pSheB comprises 91 ORFs, which cover approx. 90% of its sequence (Table 1). Over half of the identified genes constitute the backbone of the plasmid, coding for proteins involved in: replication and DNA synthesis (ORFs 1–2 and possibly ORFs 7 and 10), conjugal transfer (ORFs 27–48), and plasmid stability, including proteins of the ParA-B family partitioning system (ORFs 56–57), addiction module encoding toxin and antitoxin of the HicA-B family (ORFs 19–20), and a putative resolvase of the serine recombinase superfamily (ORF 66) which may be involved in the resolution of potential multimeric forms of circular replicons [32,33]. Interestingly, approx. 50% of the sequence of the plasmid pSheB, corresponding mainly with its backbone (region 1–41,056 nt), shares high similarity (up to 96%) with the autonomous replicons of several *S. baltica* strains (BA175, OS678, OS195, OS185, OS223), as well as the megaplasmid of *Shewanella oneidensis* MR1.

The common feature of pSheB and the abovementioned *S.baltica* plasmids is an atypical replication module structure, in which the replication initiation protein (Rep) has not yet been clearly defined. Thus, to indicate the potential Rep, the putative protein products of pSheB ORFs have been searched against the representative plasmid RepA-E,N-M proteins of *Escherichia coli* and *Staphylococcus aureus* found in the Swiss-Prot database, as well as Rep proteins of several other annotated *Shewanella* spp., including: RepA of *S. oneidensis* MR1 (acc. no. NP_720340.1) and RepB of *S. oneidensis* (acc. no. ALI93255.1). The comparative analysis, however, produced only low sequence similarity results between the annotated Rep proteins and the protein products of pSheB ORFs: 1, 7, and 10, encoding a putative DNA polymerase, endonuclease and ParB protein, respectively. Of these proteins, the product of the *parB* gene (ORF 10), identical to the ParB domain protein nuclease of *S.baltica* BA175 (acc. no. AEG13584.1), which homologs can be found also in other *Shewanella* spp. genomes, seems to be the most probable candidate for a Rep-like protein. Interestingly, the putative protein product of pSheB ORF 10 lacks its ParA counterpart, and is almost twice the size of a typical ParB protein (624 aa, compared to, e.g., 350 aa putative ParB encoded by ORF 57). This is a further suggestion that its function differs from the typical ParB proteins. Alternatively, one of the proteins forming the aforementioned ParA–B partitioning system encoded by ORFs 56–57 may serve as a replication initiator, especially as in silico analyses indicated a partition_RepA domain (TIGR03453) within the ParA protein, which is also found in RepA proteins. Moreover, the putative ParA of pSheB shows 46% similarity (limited by the presence of gaps) with the RepA protein (acc. no. AAC83387.2) encoded by the *repABC* module of *Paracoccus versutus* plasmid pTAV1 [34]. However, none other pSheB-encoded proteins show homology with the other Rep proteins of *P. versutus*. The replication system of pSheB is especially interesting, as the so far undertaken incompatibility based plasmid-curing manipulations, successfully performed for other strains isolated from the Zloty Stok mine (e.g., pSinA [35]; US Patent 9243255) did not result in its removal from the host cell. This may be caused by the presence of the aforementioned toxin-antitoxin systems, which need to be deactivated prior to plasmid-curing. Further experiments will be performed to explain the functioning of the pSheB replication system.

The conjugal transfer module of pSheB is located between nt 13,252–40,555 of the plasmid (ORFs 27–48, all with the same orientation), and it shares 92–96% sequence similarity with analogous modules found in other *S.baltica* plasmids. The genetic structure of the pSheB conjugal transfer module is concise and seems to comprise all the necessary genes. The relaxosome components are encoded by the *traD* (ORF 29)*, traH* (ORF 32)*, traI* (ORF 27)*, traK* (ORF 44)*,* and *traY* (ORF 48) genes. The genes coding for putative mating pair formation components: TraA-C (ORFs 47, 43, and 41), TraE-F (ORFs 45 and 35), TraL (ORF 46), TraN (ORF 36), TraU-W (ORFs 38, 42, and 39), as well as TrbB-C (ORFs 34 and 37), and TrbI (ORF 40) have also been identified. The putative protein product of pSheB ORF 31 shows 95% identity with the TraG protein of *S. baltica* OS223 (acc. no. ACK48883.1), which is also essential for conjugal transfer. The complete list of ORFs identified within the plasmid pSheB, with the indication of their potential function is presented in Supplementary Materials (Table S2).

The similarity of the pSheB backbone to those of *S.baltica* plasmids may be an indication that all of them derived from a common ancestor. In turn, the differences in the remaining parts of their sequences, most likely resulting from HGT and other recombination events, may be used to track their evolutionary history.

The unique feature of pSheB not only among *Shewanella* spp. plasmids, but all the known extrachromosomal replicons is the presence of two phenotypic modules providing arsenic resistance (*ars*) and arsenic respiration (*arr*) capabilities. The modules are located between 57,887 and 81,013 nt of the plasmid sequence.

The arsenic resistance module is comprised of a standard *arsDABC* operon (ORFs 74–77) and regulatory *arsR* genes localized both upstream (ORF 68) and downstream (ORF 81 and 83) of the four-gene operon core. The *arsC* gene product of *Shewanella* is a member of the cytoplasmic glutathione-glutaredoxin-dependent arsenate reductase clade, and is present in three copies. In turn, the *arsB* and *arsA* genes of O23S determine the membrane carrier and ATPase components of an arsenite efflux pump that removes arsenite from the cytoplasm. Upstream the *arsDABC* operon, *arrA* (ORF 78) and *arrB* (ORF 79) genes, encoding the two subunits of respiratory arsenate reductase are located. In the proximity of the genes which protein products are directly involved in arsenic transformations, several other genes encoding efflux transport elements are localized (ORFs 72–73, 81, 86, and 90). The *ars/arr* region of O23S is also flanked by genes coding for thioredoxins (ORFs 79 and 87), which may be involved in sulfur species transformations carried out by the strain.

**Figure 3:**
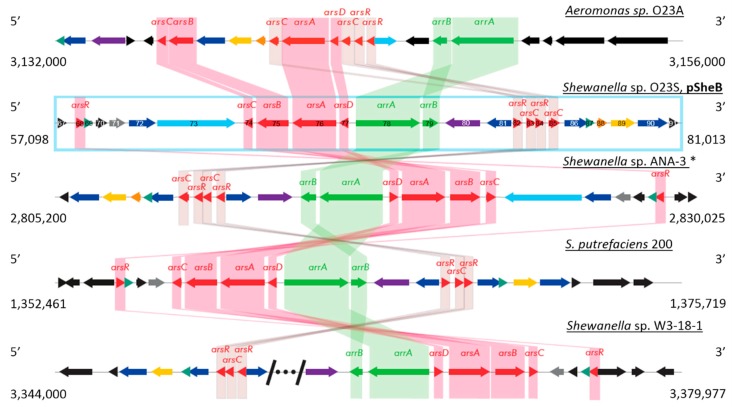
Comparative analysis of the arsenic resistance and respiration genes found within the pSheB plasmid (module within the frame, pSheB open reading frame (ORF) numbers are given), with the arsenic respiration (*arr*) and resistance (*ars*) modules of *Aeromonas* sp. O23A (top) and the most similar modules found in genomic databases. Genes encoding proteins involved in arsenic resistance and arsenic respiration ability are colored in red and green, respectively. Genes encoding proteins with similar function are color-coded: cytC—light grey; g3p—yellow; glutathione synthetase—purple; phosphatase—orange; permease—dark blue; other efflux system components—light blue; grey-green—redox proteins. Phage region in *Shewanella* sp. W3-18-1 was omitted as indicated by /^…^/ sign. * *Shewanella* sp. ANA-3 was selected to represent the *ars* and *arr* modules of *S. putrefaciens* CN-32 with almost identical structure.

Comparative analysis of the *ars/arr* region of O23S with other, most similar gene modules involved in arsenic transformations found in the genomic databases using BLAST is shown below (Figure 3). It was found that the *arr* module is almost identical to other *arr* modules found in several *Shewanella* spp. strains: *Shewanella* sp. ANA-3 (94%; acc. no. CP000469.1), *Shewanella putrefaciens* CN-32 (94%; acc. no. CP000681.1), *S. putrefaciens* 200 (86%; acc. no. CP002457.1), and *Shewanella* sp. W3-18-1 (86%; acc. no. CP000503.1). Interestingly, the arsenic modules found within the latter strain are separated by a phage region of approx. 13 kbp, of which some coding sequences show similarity to a Mu phage, including genes encoding putative transposases A and B (acc. no. ABM25750.1 and ABM25751.1, respectively). This may suggest that arsenic related genes may be affected, and possibly transferred via phage infection.

However, it must be emphasized that for all these strains the *arr* module is located within the chromosome, not in extrachromosomal location. For this reason, pSheB is the first known natural plasmid comprising both *arr* and *ars* modules. Moreover, pSheB carries the only copy of dissimilatory arsenate reductase genes in the genome of O23S what is a unique feature of this strain compared to other DARB.

The comparative analysis showed that co-localization of the *arsDABC* operon with arsenic respiration genes *arrA-B* is common in DARB. However, while the general structure of the *ars/arr* region is conserved in at least several members of *Shewanellaceae*, due to genome rearrangements, and especially homologous recombination, the structure of the module may be different without affecting the functionality, as shown earlier for *Aeromonas* sp. [9]. The common isolation place of two DARB species, *Shewanella* sp. O23S and *Aeromonas* sp. O23A, and the fact that genome of both these strains has been sequenced enabled comparative analyzes between them. O23A was the first described DARB strain belonging to this genus, showing also interesting properties of iron–arsenic mineral dissolution, both directly and indirectly—due to siderophore production, but also potential for increased adherence factor and biofilm production, which in turn may play a role in entrapment (immobilization) of arsenic and other heavy metal ions within its structure [4]. As it has been previously shown by Drewniak et al. [10], *Shewanella* sp. O23S shares these properties, being capable to directly and indirectly mobilize arsenic from rocks and minerals. This study also confirmed the capability of O23S to adhere to surfaces (see Results 2.4), which—together with the ability to simultaneously reduce arsenic and (thio)sulfate species to form an insoluble precipitate—indicates the role of the strain in As immobilization. At genomic level the strains share similarity with respect to genes involved in arsenic transformations, but the analyses also revealed several interesting differences between them.

Firstly, the localization of the arsenic respiratory genes (*arrA* and *arrB*) in the strains differs. In O23A copies of these genes can be found on chromosome only, whereas in O23S an *arrA-B* module is located on the plasmid pSheB. In fact, this *arr* module localization is a unique feature of O23S, being the host strain of the plasmid pSheB. To date, pSheB remains the only known natural vector of arsenic respiration genes, and the plasmid itself, as well as its functional derivatives, has already acquired patent protection. The plasmid pSheB is also a natural vector of *ars* genes, but this particular feature is shared with the plasmid pSinA, also isolated from a strain originating from the Zloty Stok mine (*Ensifer* sp. M14) [3].

Secondly, the genomic context of the arsenic respiration and resistance modules in O23S is more complex than in O23A. Analyses indicated that O23S carries more copies of non-respiratory arsenic reductases (*arsC*), both on the chromosome and on the plasmid pSheB than O23A. This, together with the potential difference in overall metabolism and gene expression, may explain the difference in arsenate reduction kinetics between the strains: O23S is capable of complete reduction of 200 umol As(V) (at a concentration of 2.5 mM) within just 4 h, whereas complete reduction of such amount of arsenates under the same conditions was not observed for O23A, neither in 24, nor 48 h. This may also be an indicator that arsenate reduction carried out by O23A is to some level reversible, whereas O23S activity does not result in such a phenomenon. However, regardless of the differences in putative *arsC* gene copy number, both the strains show remarkably high resistance to arsenic, indicating that these systems provide efficient protection against the toxic effect of this metalloid.

To confirm the functionality of the products encoded by the *arrA* and *arrB* genes of pSheB and their role in dissimilatory arsenate reduction the *arr* module (coordinates: 65,155-68,435) was cloned into the vector pBBR1-MCS2 (Kmr), in the *Escherichia coli* TOP10 strain, which was then grown on minimal medium with arsenate as the sole electron acceptor and lactate as an electron donor and carbon source. The reduction of arsenates to arsenites by the obtained *E. coli* MR1 (pARR1A) strain, was qualitatively assessed by HPLC. The analysis indicated that after 120 h of cultivation of the *E. coli* MR1 (pARR1A) strain, arsenites were present in the medium. In the control variant with the *E. coli* TOP10 strain (without the *arr* module), carried out in identical conditions, reduction of As(V) to As(III) was not observed. This experiment confirmed the role of the *arr* module of pSheB in dissimilatory reduction of arsenates and indicated its functionality also in a heterologous host.

In order to verify the functionality of the entire *ars* and *arr* modules of O23S comprised within the 57,000–81,000 nt of the plasmid sequence, a pool of clones from pre-prepared fosmid libraries was used. Appropriate fosimids carrying the modules were selected on the basis of probing using specific primers, followed by sequencing and sequence mapping within the *Shewanella* sp. O23S genome. The selected clones with genes potentially encoding interesting phenotypic traits within the phenotypic module were grown under aerobic and anaerobic conditions to determine whether and to what extent the cloned plasmid fragment precluded the appearance of increased clone resistance to arsenic. Based on the performed minimal inhibitory concentration (MIC) analyses, clones showing arsenic resistance (above 10 mM AsV) were selected with simultaneous ability to reduce arsenates or lack thereof. The AgNO_3_ test was used to assess the arsenic reduction ability (data not shown). The *E. coli* F96401-1 strain harboring fosmid 41C comprising the complete *ars/arr* region of the pSheB plasmid, was characterized by increased resistance to this metalloid as compared to the *E. coli* F96401-1 strain (Table 2 and Table 3).

As shown above, the *E. coli* strains used as controls encoding only the *arsDABC* operon, but without the *ars/arr* region of O23S, are resistant to only up to 100 mM of As(V) and 5 mM of As(III), whereas the strain with the fosmid 41C showed resistance to up to 350 mM of As(V) and 8 mM of As(III). The results of MIC analyses confirm the functionality of the resistance module of pSheB in the heterological host strain. Therefore the *ars/arr* region of O23S, following its transformation to other strains, may be potentially used to increase arsenic resistance of the host.

The native O23S strain is resistant to 500 mM of As(V) and up to 10 mM of As(III), what may indicate the presence of other mechanisms of resistance in the O23S strain, e.g., other inorganic ion transport systems or additional components of the *ars* module present in O23S genome which may further increase arsenic resistance. In fact, comparative analyses indicated additional copy of the *ars* operon in the chromosome (ORFs 340–343) comprising, i.a., genes for arsenic resistance protein (ArsH), arsenate reductase (EC 1.20.4.1) and arsenic efflux pump protein, as well as resistance operon repressor and other regulatory proteins (ArsR). Moreover, the module also comprises a gene for a putative MFS-type efflux pump, possibly capable of transporting 1-arseno-3-phosphoglycerate out of the cell. Therefore, this chromosome-encoded system may additionally facilitate arsenic removal from the O23S cells, thus increasing its resistance.

#### 2.2.3. Prophages and Plasmid-Like Phages

The genome of *Shewanella* sp. O23S has been searched for putative prophage sequences using the PHAST program. The analysis revealed the presence of three such regions, named MuSsp1_O23S (coordinates 1,029,333-1,053,277), MuSsp2_O23S (coordinates 1,713,353-1,753,000) and LambdaSsp¬_O23S (coordinates 5,032,534-5,066,235). The first two were very similar to MuSo1, and MuSo2 of *Shewanella oneidensis* MR-1 prophages [36] and also shared genome organization and sequence relatedness with *E. coli* transposable phage Mu [37] and Mu-like phages and prophages. The proteome of LambdaSsp¬_O23S did not share any homologues with LambdaSo of *S. oneidensis* MR-1, but showed similarities with prophage sequences presented in other *Shewanella* spp., e.g., *Shewanella baltica* OS195 (acc. no. CP000891). To verify whether the recognized prophage regions are active phages, the cells of *Shewanella* sp. O23S were treated with mitomycin C. This approach caused the induction of only the MuSsp1_O23S prophage. Its nucleotide sequence was confirmed by resequencing of DNA isolated from the CsCl-purified viral particles.

Sequence analysis showed that the genome of the MuSsp1_O23S phage was 37 883 bp DNA with a G+C content of 47.9%, and comprised 45 ORFs. The amino acid sequences of all the putative proteins were compared with the known sequences using the BLAST program. Twenty five of these 45 protein coding genes were assigned to putative protein functions. The functions of the remaining 20 putative protein coding genes remained unknown and they were annotated as hypothetical proteins. The majority of MuSsp1_O23S ORFs were oriented in the same direction with the exception of the putative c repressor (ORF1) and ORF28 that were transcribed in the reverse direction. Positions, sizes and putative functions of the proteins are listed in Supplementary Materials (Table S3).

Thirty nine putative protein products of the MuSsp1_O23S phage genes showed at least 46% identity with the proteins encoded by MuSo1 of *S. oneidensis* MR-1. Only 6 ORFs: 13 (Clp protease), 15 (hypothetical protein), 29–31 (transcriptional regulator, reverse transcriptase and hypothetical protein) and 41 (hypothetical protein) were unique for MuSsp1_O23S. It is worth emphasizing that MuSo1 was not capable of forming infectious particles probably due to the effects of several transposition events within its genome [36,38]. Comparative analysis of genomes of both phages revealed two insertions of transposase genes into the regions of MuSo1 corresponding to the location of ORF14-16 and ORF40-42 of MuSsp1_O23S. As mentioned above, the middle ORFs are absent in the MuSo1 genome.

In contrast to MuSsp1_O23S, the second identified Mu-like prophage sequence, MuSsp2_O23S did not seem to be an active prophage element. We also were unable to obtain viral particles of LambdaSsp¬_O23S.

TEM analysis showed that the virion of MuSsp1_O23S had an icosahedral head about 60 nm in diameter and a tail about 230 nm long and 12.4 nm wide. These morphological features indicate that this phage belongs to the *Siphoviridae* family (Figure S2). As we could not use TEM analysis for a direct classification of the MuSsp2_O23S and LambdaSsp¬_O23S prophages, VIRFAM analysis was used instead. VIRFAM predicted MuSsp2_O23S to be a member of the *Myoviridae* of Type1, clustered with *Enterobacteria* phage P2, Vibrio phage CTXphi and *Haemophilus* phage HP1 (Cluster 9). VIRFAM also predicted LambdaSsp¬_O23S to be a member of the *Siphoviridae* of Type 1 clustered with *Enterobacteria* phage Mu, Pseudomonas phages D3112 and M22, and *Burkholderia* phage Bcep (Cluster 8).

Apart from the prophages integrated with the O23S chromosome other phage-like elements have been identified by sequencing analysis, i.e., pSheC (8147 bp), pSheD (6658 bp), and pSheE (14805 bp), which seem to replicate extrachromosomally. They all show similarities with episomally replicative filamentous phages (Tables S4 and S5). The following regions of pSheC and pSheD exhibited high sequence similarity: (i) 4373–7143 of pSheC and 3077–5820 of pSheD, 75% identity; (ii) intergenic regions: 886–1059 of pSheC and 667–828 of pSheD, 70% identity. This also applies to the putative protein products encoded in the first two sections. Of 11 proteins, encoded by pSheC and pSheD, five share sequence similarities (identity between 42 and 78%, see Tables S4 and S5). Five of the putative protein products are also analogues of the well characterized F-pilus specific *E. coli* phages known as Ff-group [39] for which predicted functions are: minor and major virion proteins (counterparts of Ff-like proteins pVII, pVI and pVIII), receptor-binding and assembly proteins (pII and pI).

On the other hand, putative replication proteins of pSheC and pSheD do not share amino acid sequence similarities. However, ORF 4_SheD and ORF 6_SheD proteins are homologues to the replication (endonuclease) and ssDNA binding proteins of Ff-like phages, respectively (Table S5). Whereas, ORF 5_SheC and ORF 6_SheC proteins are homologues to the replication-associated protein RstA and integration (lysogeny)-associated protein RstB of Vibrio phage CTXphi, respectively (Table S4). ORF 5_SheC and ORF 6_SheC seem to be also counterparts of RstA and RstB-like proteins of the low-temperature-inducible phage SW1 of *Shewanella piezotolerans* WP3 (56% and 71% identity, respectively). It is worth emphasizing that SW1 can exist as an extrachromosomal plasmid or integrate into the chromosome same as pSheC (see below).

Interestingly, sequence analysis of these plasmids revealed that pSheE is simply a combination of pSheC and pSheD molecules which are also present in the cell as autonomous replicons. Moreover, a copy of pSheC exists within the O23S chromosome (coordinates 2,510,258–2,518,404). Upstream the start of the chromosomal copy of pSheC a region identical to the last 17 nucleotides of pSheC was found (AAAGATGCGCACAATGT, coordinates 2,510,241–2,510,257). This could be the attachment site of the host genome (attB) for the pSheC element. Almost identical sequence (one mismatch, underlined, AAAGCTGCGCACAATGT) is present in pSheD (last 17 nucleotides, coordinates 6642–6658). Possibly, these sequences are involved in the production of a fusion form of pSheC and pSheD, i.e., pSheE.

To experimentally confirm that all three plasmids (and in particular pSheE) are present in O23S cells, we conducted PCR reactions with combination of four starters and total DNA isolated from O23S cells as a template (Figure S3). As we obtained PCR products of expected size in each probe therefore we concluded that not only pSheC, pSheD, but also pSheE indeed exist as extrachromosomal plasmids.

#### 2.2.4. Other Mobile Genetic Elements

Other mobile genetic elements found within the O23S genome, apart from prophages include several putative insertion sequences with transposases of the IS116/IS110/IS902 (encoded by ORFs 102 and 3350) and VIBHAR_02188 (ORFs 701, 1543, 2201, and 2252) families, as well as InsN and InsO transposases for IS911 (ORFs 1811–1812, 1971, 2002, 2006, 2086–2087, 2095, 2249, 3947). Genes encoding putative transposases of IS3E (ORF 2108), ISSod1 (ORF 3784), as well as the OrfA transposase were also found in the O23S chromosome. Interestingly, in the genome of O23S genes coding for potential HflC (ORF 632) and HflK (ORF 631) proteins were identified. Counterparts of these two proteins encoded by *E. coli* help to govern the stability of phage lambda CII protein by inhibition of the HflB (FtsH)-mediated proteolysis, and thereby influence on the lysogenization frequency of phage lambda, and possibly—also of the phages encoded in the O23S genome.

### 2.3. Other Heavy Metal and Antibiotic Resistance Genes

The *Shewanella* sp. O23S genome was also analyzed for genes possibly involved in resistance to inorganic ions. Based on the analyses several other heavy metal resistance genes were found, including genes encoding cobalt-zinc-cadmium resistance protein CzcD (ORF 2365), magnesium and cobalt efflux protein CorC (ORF 3465), Mg/Co/Ni transporter MgtE (ORFs 3511 and 3944), Co/Zn/Cd efflux system proteins of the CzcABC family (ORFs 555–557, 3994, 4561 and 4753–4755). Additionally, O23S chromosome encodes a chromate transport protein ChrA (ORF 914) and a putative lead, cadmium, zinc, and mercury transporting ATPase (ORF 1494), as well as molybdenum ABC transporter components (ORFs 3755–3757) and copper/silver efflux RND transporter CusA-B proteins (ORFs 4465–4466). The functionality of the indicated resistance genes was previously assessed in MIC analyses by Drewniak et al. [10]. Further analyses were carried out to investigate the effect of co-presence of these heavy metal ions and arsenic—to investigate the functionality of the resistance mechanisms in natural environment-like conditions, where arsenic is constantly present. The growth tests were performed in MS medium, also in a variant with the addition of 10 mM Tris-HCl buffer to increase bioavailability of the metals. The results are shown in (Table 4). The functionality of other, non-specific resistance mechanisms—including efflux pumps, numerous copies of which have been found to be encoded by the O23S genome, was tested in MIC analyses for phosphates, glycerol, and NaCl. In addition, the effect of both forms of arsenic, i.e., As(III) and As(V), on the functionality of these mechanisms was tested. The results are shown in (Figure S4).

It was found that on minimal medium O23S is capable of growth in the co-presence of arsenic and 1 mM of either iron, nickel, or zinc. Of these variants, most intensive growth in the presence of both 2.5 and 5 mM of As(III) was noted for iron and nickel. In turn, in the co-presence of As(V) and iron, O23S growth was limited. The growth on copper was highly limited even without the co-presence of arsenic, indicating that the efficiency of resistance mechanisms is low. Nevertheless, the obtained results, together with those from the previous MIC analyses, confirm the functionality of the indicated resistance genes. Moreover, the simultaneous presence of arsenic and phosphates (similar in structure to arsenates) did not hinder the growth of the strain even in as high concentrations of phosphates as 2000 mg/L. Interestingly, the presence of even 1% of glycerol, which is known to disturb the structure and thus the functionality of cell membrane components including efflux/transport proteins turned out to be toxic to O23S, when As(III) was also present in the medium. This result indicates that the effective transport of the As(III) ions out of the cell is crucial for O23S resistance.

This is also true for the other resistance mechanisms identified in the genomes (e.g., *czcA-B*) which provide resistance to other heavy metals and which functionality has also been confirmed. In fact, apart from the copies of arsenic-transporting pumps a large number of genes in the O23S genome encoding potentially functional efflux pumps characterized by low specificity (broad range of exported molecules/chemical species) have been found. These may provide versatile means of exporting potentially toxic species/metabolites out of the O23S cells. As it was found these resistance mechanisms remain functional in the simultaneous presence of arsenic and other metals including copper and nickel ions. Their functionality was only limited by the co-presence of arsenic and zinc, and at very high concentrations of glycerol which may cause a disturbance in the structure of membrane proteins. Given all the above, *Shewanella* sp. O23S seems to be well suited for growth in highly contaminates areas.

### 2.4. Antibiotic Susceptibility and Putative Antimicrobial Drugs Resistance Mechanisms of O23S

Apart from specialized metal ion transporters, in the chromosome of O23S multiple proteins coding for multidrug (MdtK/NorM (MATE family) of MDR efflux pumps, Bcr/CflA and MtrF family efflux proteins, possibly involved in antibiotic resistance were found. Among these at least 17 copies of multidrug efflux system, inner membrane proton/drug antiporter MexF (RND type) of MexEF-OprN system and numerous RND efflux system, inner membrane transporter were identified. In addition, based on in silico analyses it may be possible to predict O23S resistance to (i) macrolides: ORF 3752 encodes putative macrolide export ATP-binding/permease protein MacB; ORF 3752—macrolide-specific efflux protein MacA, (ii) acriflavin (e.g., ORF 880), mitomycin (ORF 520) and microcin (McbG-like protein encoded by ORF 4045). Moreover O23S carries several genes encoding putative class C beta-lactamases (EC 3.5.2.6) and penicillin binding proteins (PBPs). The functionality of the abovementioned genes and other potential antibiotic resistance genes identified within the O23S was assessed by E-test MIC analyses. The MIC levels were read according to EUCAST recommendations after approx. 20 h of incubation and were additionally verified after 48 h (as the strain is different from those described by Eucast). *E coli* strain 92025 was used as a susceptibility control. The results of the antibiotic susceptibility analyses for O23S are shown in Table 5.

On the other hand genes for invasin (encoded by ORFs 2623 and 4064), which play a role in promoting entry during the initial stage of infection, vibriolysin (ORFs 2096 and 4144) and secreted microbial collagenase (ORF 3991) were found. These lytic enzymes, and particularly the microbial collagenases (EC 3.4.24.3) are strongly linked to bacterial pathogenesis [40]. In turn, genes for colicin V (ORF 2883; identical to bacteriocin production protein of *Shewanella* sp. WE21; acc. no. RBP77813.1), a peptide antibiotic that kills sensitive cells by disrupting their membrane potential once it gains access to the inner membrane from the periplasmic face, as well as holin-like protein CidA (ORF 3603) and murein endopeptidase (ORF 3600; identical to *S. putrefaciens* protein acc. no. RBP81984.1) have also been identified. Other genes which protein products may be involved in pathogenicity include: putative hemolysin encoded by ORFs 730, 2075, 2435, and 3416.

Genome analysis results, confirmed by physiological experiments indicate that the use of *Shewanella* sp. O23S in bioremediation technologies, e.g., dedicated to controlled removal of arsenic from solid matter does not pose serious biosafety risks. Among the antimicrobial agents tested only for trimethoprim O23S showed increased resistance, but only after twice longer incubation than recommended in the standard EUCAST procedure. However, to limit the spread of potential antimicrobial resistance genes in the environment, genetic modification of the O23S strain or, alternatively the use of another strain devoid of such genes, but capable of acquiring and stable maintenance of the plasmid pSheB, may be required. Moreover, as it was shown in the patent, it is possible to construct a new vector carrying the gene module coding for the proteins involved in dissimilatory arsenate reduction and/or resistance to arsenic. Such vectors, devoid of potential environmentally unsafe genes can be used to obtain strains with increased bioremediation potential. Care must be taken, however, to reduce the risk of uncontrolled dissimilatory reduction.

### 2.5. Adherence and Biofilm Formation Analysis

The surface adherence and biofilm production capabilities of *Shewanella* sp. O23S were assessed using the microplate method in which the optical density of both the entire liquid culture and the adherent cells (following staining) is measured. After the first 24 h of growth on LB medium under aerobic conditions, OD_600 nm_ of the O23S culture increased from the initial 0.06 ± 0.01 (inoculation) to 0.71 ± 0.02, and fluctuated between 0.54–0.78 during the next 48 h0 (Figure S5). On media supplemented with 5 mM of As(III) or As(V), the growth of the strain was noticeably slower, as indicated by the t = 24 h OD_600 nm_ values: 0.042 ± 0.01 and 0.48 ± 0.02, respectively. In turn, the highest OD_570 nm_ value, indicating the most significant number of adherent cells in the culture was observed for the LB+As(V) variant (0.72). This suggests that increased surface adhesion and biofilm production may be beneficial in the presence of toxicants such as arsenic. However, as indicated before by Drewniak et al. [41], biofilms may tend to accumulate metabolites, including the metal ions removed from the bacterial cells, thus likely increasing the local concentration of the toxic species, making the adherent-type lifestyle unfavorable. This effect may explain the significant decrease in the OD_570_/OD_600_ ratio for both the variants with arsenic (Table S6). Interestingly, the 4-times decrease in OD_570 nm_ for the LB+As(V) variant after 48 h did not result in an as significant increase in OD_600 nm_ value which is correlated with the number of non-adherent cells. This may suggest that the adherent cells lysed or caused the lysis of some of the free-living cells. For the other variants, the decrease in OD_570 nm_ throughout the experiment, regardless of the changes in OD_600 nm_, was also observed. This indicates that the depletion of nutrients is the most likely the second-main cause of changing of O23S lifestyle towards a culture consisting mostly of free-living cells.

### 2.6. Metabolic Preferences of O23S

Metabolic preferences of *Shewanella* sp. O23S in terms of capability of using different compounds as C, N, P or S sources, were investigated using Biolog^TM^ MicroArray assay. The changes in OD_600 nm_ in time, including the length of the lag phase, as well as the total area beneath the growth curve were examined. For carbon sources, the analysis was complimentary to the preliminary tests performed on Biolog^TM^ *EcoPlates* by Drewniak et al., during which 31 carbon sources were tested [10]. Apart from the 18 previously recognized substrates (including lactate, citrate, and acetate, for which also arsenic reduction kinetics were shown) O23S was found to readily utilize succinic acid, D-gluconic acid, maltose, sucrose, uridine, maltotriose, fumaric acid, (cyclo)dextrin and, to lower extent, several other compounds as the sole carbon sources. In turn, growth analyses carried out on Phenotypic MicroArrays amended with nitrogen sources (PM3B) showed that the strain can obtain nitrogen from a number of amino acids and nucleosides, and all the tested dipeptides. Interestingly, O23S can also utilize ammonia and glucuronamide as nitrogen sources, with a similar growth rate to that on amino acids and their derivatives. Biolog™ analysis, however, did not confirm that *Shewanella* sp. O23S can utilize nitrate or nitrite as the sole sources of nitrogen. In turn, the strain was shown to utilize numerous compounds as phosphorous sources, including phosphorylated nucleotides and nucleosides, as well as esters of amino and phosphoric acids. O23S was also found to utilize two inorganic compounds as the sole sulfur sources, i.e., thiosulfate and thiophosphate, and several organic compounds, including lanthionine, glutathione, and derivatives of methionine and cysteine. The results of the Biolog^TM^ analysis are shown in Supplementary Materials (Figures S6–S9).

## 3. Materials and Methods

### 3.1. Strains, Vectors, Media, and Growth Conditions

The investigated *Shewanella* sp. O23S strain was isolated from effluent waters and bottom sediments of dewatering systems from the Zloty Stok gold mine (SW Poland) by Drewniak et al. [42] and has been deposited in The Bank of Biological Materials at the Institute of Biochemistry and Biophysics, Polish Academy of Sciences. The strain was grown in liquid cultures in Luria-Bertani (LB; [43]) or minimal salt (MS) medium (containing in g/L: NaCl, 1.17; KCl, 0.30; NH_4_Cl, 0.15 ; MgCl_2_∙6H_2_O, 0.41; CaCl_2_∙2H_2_O, 0.11; KH_2_PO_4_, 0.20; NaHCO_3_, 2.00; Na_2_SO_4_∙10H_2_O, 0.28), supplemented with 2 ml/l of trace metals solution [44], 5 mM lactate, and, where necessary, 2.5 mM sodium arsenate and yeast extract (0.04% *w/v*) as a source of vitamins, at room temperature (26 ± 2 °C). *Escherichia coli* F96401-1 strains, hosting fosmids with the fragments of O23S genome (fosmid genome library, see below Section 3.2.2) were cultivated in the same media amended, where necessary, with arabinose promoter inductor (L-arabinose), at the final concentration of (10 µg/mL), at the temperature of 37 °C. *E. coli* TOP10 was used as the host of constructs with the O23S arsenic respiration module. *E. coli* strain 92025 was used as susceptibility control in antibiotic susceptibility tests.

### 3.2. DNA Manipulations

#### 3.2.1. DNA Isolation

Standard DNA manipulation methods were performed as described by [43]. Total DNA was extracted using a kit (Genomic Mini, A&A Biotechnology, Poland) from bacterial cells harvested by centrifugation of an overnight culture carried out in LB medium. Purified DNA was used as the template for genome sequencing. Fosmid DNA was isolated analogously or using a kit (Plasmid Mini, A&A Biotechnology, Poland), whereas (mega)plasmid DNA was isolated by CsCl gradient method [43]. The obtained plasmid DNA was separated by 0,8% *v/v* agarose gel electrophoresis visualized by EtBr staining.

#### 3.2.2. Genome Sequencing, Assembly and Analysis

Genome assembly was performed using a combination of Illumina short data reads and PacBio RSII long reads. Pair-end library with an average insert size of 460 bp was prepared using the Illumina TruSeq v2 kit and sequenced on an Illumina HiScan with 2 × 100 nt read length. Illumina sequencing yielded 1968 million reads, which were first trimmed to remove adaptor sequences using cutadapt (v1.8) and then quality trimmed using sickle2 (mean quality 30, min. length 50 nt). PacBio sequencing data was obtained from the Museum and Institute of Zoology, Polish Academy of Sciences. Library sequencing with an average insert size of 20 kb resulted in 184,284 reads with the mean length of 1.88 kb (longest read: 49.77 kb). Assembly of the obtained reads was performed using SPAdes (v.3.8.0)—careful option with PacBio reads supplied as filtered subreads fasta. This yielded 24 scaffolds sequences, with a N50 length of 791 kb (longest scaffold—1869.18 kb). The remaining gaps and sequence errors in the genome assembly were verified by the PCR amplification of DNA fragments and restriction digestion of the fosmid genome library of O23S created in pNGS FOS (Lucigen) with an average insert size of 38 kb. Fosmid clones and PCR products were sequenced by Sanger sequencing with an ABI3730xl Genetic Analyzer (Life Technologies, USA) using BigDye Terminator Mix v. 3.1 chemistry (Life Technologies, USA). All of the sequence errors and misassemblies were further corrected using Seqman software (DNAStar, USA) to obtain complete nucleotide sequence of bacterial genome.

The complete nucleotide sequences of plasmids pSheC-E were determined at the DNA Sequencing and Oligonucleotide Synthesis Laboratory (oligo.pl) at the Institute of Biochemistry and Biophysics, Polish Academy of Sciences. High-throughput sequencing of the MID-tagged shotgun plasmid-library was performed using an FLX Titanium Genome Sequencer (Roche/454 Life Sciences). Newbler de novo assembler software (Roche) was used for the sequence assembly. Final gap closure and sequence polishing were performed by capillary sequencing of PCR products using an ABI3730xl DNA Analyzer (Applied Biosystems).

The obtained O23S chromosome and plasmid sequences was automatically annotated using RAST [45] and selected open reading frames (ORFs) were verified manually using Artemis software [46] and BLAST programs [47] provided on the National Center for Biotechnology Information (NCBI) website (http://blast.ncbi.nlm.nih.gov/Blast.cgi). Plasmid nucleotide sequences were also automatically annotated using the RAST server and the resulting annotations were then thoroughly manually curated using Clone Manager (Sci-Ed8) and Artemis software. Similarity searches were performed using the BLAST programs, UniProt (http://www.uniprot.org/) and REBASE [48] databases.

#### 3.2.3. Phage Induction, Annotation and Analysis

Prophage sequences within the *Shewanella* sp. O23S genome were identified using PHAge Search Tool (PHAST) [49] available at http://phast.wishartlab.com/. To induce potential prophages, bacterial cells were treated with mitomycin C. The resulting lysate was purified by PEG precipitation and CsCl density gradient separation. The visible band was collected and analyzed for the presence of phage particles by transmission electron microscopy (TEM). Genomic DNA of the phage was extracted from CsCl-purified viral particles and was subjected to high throughput resequencing. The obtained phage sequence was automatically annotated using the RAST server and the resulting annotations were then thoroughly manually curated. BLASTP and Psi-BLAST algorithms were used for the similarity searches in the NCBI, UniProt and Pfam (http://pfam.xfam.org/) databases. A phage family search was carried out using VIRFAM [50]. The manually annotated sequences of O23S replicons pSheA-E were deposited in the GenBank database.

### 3.3. Physiological Analyses

#### 3.3.1. Resistance to Phosphate, Glycerol, and Heavy Metal Ions

Functionality of the genome-encoded resistance mechanisms of O23S was analyzed by determining minimal inhibitory concentrations (MICs) of various substances, also in the co-presence of arsenite or arsenate ions. The experiments were carried out in 96-well microplates containing either MS medium, with or without the addition of 10 mM Tris-HCl buffer or modified LB medium. Depending on the variant, the media contained different concentrations of: (i) phosphate: 0–0.2%, (ii) glycerol: 0–20%, (iii) NaCl: 0–3.0%, and (iv) heavy metal salts: NaAsO_2_, Na_2_HAsO_4_, CuSO_4_·5H_2_O, FeCl_3_, NiCl_2_, and ZnSO_4_·7H_2_O. Analytical grade chemicals were used. The MIC was defined as the lowest concentration of each compound that completely inhibited bacterial growth. Unless stated otherwise, in all the above experimental variants the pH of the medium was 7.0 ± 0.1, and the cultures were incubated at the temperature of 26 °C. Each well of a microplate was inoculated with cells from an overnight culture to a final optical density at 600 nm (OD_600 nm_) of 0.06 (approx. 10^6^ cells/mL), which was measured using an automated plate reader (Sunrise, TECAN). Uninoculated media were used as the control. The cultures were propagated for 48 h or 96 h, and OD_600 nm_ was checked every 24 h. All experimental variants were carried out in triplicate.

#### 3.3.2. Antibiotic Susceptibility Tests

Antimicrobial susceptibility patterns of *Shewanella* sp. O23S were obtained by determining MIC for selected antimicrobial agents using Etest™ (Liofilchem, Roseto degli Abruzzi, Italy). The following antibiotics were used: aminoglycosides—gentamicin (CN; concentration of antibiotic: 0.064–1024 µg/mL), β-lactams (penicillin derivatives)—ampicillin (AMP; 0.016–256 µg/mL), β-lactams (cephalosporins)—cefixime (CFM; 0.016–256 µg/mL), β-lactams (cephalosporins)—cefotaxime (CTX; 0.016–256 µg/mL), β-lactams (cephalosporins)—ceftriaxone (CRO; 0.016–256 µg/mL), fluroquinolones—ciprofloxacin (CIP; 0.002–32 µg/mL), fluroquinolones—moxifloxacin (MXF; 0.002–32 µg/mL), phenicols—chloramphenicol (C; 0.016–256 µg/mL), ryfamicins—rifampicin (RD; 0.016–256 µg/mL), tetracyclines—tetracycline (TE; 0.016–256 µg/mL). The analysis was conducted according to the European Committee on Antimicrobial Susceptibility Testing (EUCAST) recommendations for 20 ± 2 h, at the optimal growth temperature of the strain (26 °C). *Escherichia coli* strain 92025 was used as susceptibility control. Due to slower growth of O23S compared to the EUCAST reference strain, incubation time was increased and MIC was read again after 48 h. After incubation, plates were photographed, MICs were defined and the results were interpreted according to the EUCAST breakpoint table (version 8.0). All tests were performed in duplicate.

#### 3.3.3. Metabolic Substrate Preferences (Biolog™) Test

The carbon metabolism of the *Shewanella* sp. O23S strain was previously characterized by the community level physiological profiles (CLPPs) using Biolog™ EcoPlate [10]. More detailed analyses of metabolic substrate preferences of *Shewanella* sp. O23S, were performed using Biolog^TM^ Phenotypic MicroArrays (PM) amended with various carbon (PM1, PM2A), nitrogen (PM3B), phosphorus and sulfur sources (PM4A). Each well of the plates was inoculated with 100 μL of bacterial suspension and incubated under aerobic conditions for 48 h at a constant temperature of 26 °C. OD_600 nm_ was determined using a Biolog™ reader (Biolog, Hayward, CA, USA). All plate tests were carried out in triplicate.

### 3.4. Functionality of Arsenic Resistance and Arsenic Respiration Modules

Cultures of individual *E. coli* F96401-1 strains (2000) containing the fosmid gene library of O23S were pooled into 240 groups, which were then searched for *arr* and *ars* genes by PCR, using the previously described starters [51]. The obtained products were visualized by agarose gel electrophoresis, followed by EtBr staining. The indicated clones were then analyzed both by PCR and through cultivation on MS medium described in the Section 2.1. Functionality of the *ars* module was tested in a series of MIC analyses in titration plates, in LB medium amended with appropriately diluted stock solutions of sodium arsenate (0–100 mM) or sodium arsenite (0–10 mM) salts, depending on the variant. Bacterial growth was assessed by measuring the changes in OD_600 nm_ of the cultures compared to the non-inoculated controls using an automated plate reader (Sunrise, TECAN). Measurements were carried out at 24 h intervals. Arsenic reduction was assessed following anaerobic cultivation of strains in MS medium using the test with AgNO_3_, as previously described by Drewniak et al. [10].

In order to clone the *arr* module, amplification of a DNA fragment of the size 8634 bp (comprising the region with ORF 78-79) was performed on a DNA template of the plasmid pSheB, isolated by alkaline lysis. The following oligonucleotides were used as primers: She_Mph1103F: GAAATCTTGCAGTAGCGATGCATC [position in the sequence of the plasmid pSheB: 63,978–64,001; the underlined sequence is the restriction site recognized by the enzyme Mph1103I], and She_XmaJR: *GTTGTTCCTAGG*CTGGTGCCATATCAACCTCTAG (position in the genome of the plasmid pSheB: 72,578–72,599; the sequence written in italic is an added sequence; the underlined site is recognized by the restriction enzyme XmaJI). Phusion® High-Fidelity DNA Polymerase (Thermo Scientific, Waltham, MA, USA) was used for the amplification reaction. The obtained PCR product was cloned into pBBR1MCS-2 broad-host range vector [52] in *E. coli* TOP 10 strain. The dissimilatory arsenate reduction capabilities of the obtained *E. coli* MR1 (pARR1A) strain were tested in growth experiments carried out in minimal R1-R2 medium enriched with 2 mM sodium arsenate and 5 mM sodium lactate and supplemented with 0.004% yeast extract. The culture was carried out for 120 h under anaerobic conditions (in CO_2_:N_2_ atmosphere) at 37 °C. After five days of culture, the test with 0.1 M solution of silver nitrate was carried out along with qualitative analysis of arsenic speciation by HPLC. *E. coli* TOP10 strain (without the plasmid), was used as the control.

### 3.5. Microplate Adherence Assay

Microplate adherence assay was performed as described before by Uhrynowski et al. [4]. Briefly, *Shewanella* sp. O23S was cultivated at 26 °C for 24, 48, and 72 h on separate 96-well plates in LB medium, amended where necessary with 5 mM of either sodium arsenite or sodium arsenate. After each incubation period bacterial growth was analyzed by OD_600 nm_ measurements using an automated plate reader (Sunrise, TECAN). Subsequently, the medium with non-adherent bacterial cells was removed, the plate was rinsed 3 times with water, dried, and the adherent cells were stained for 10 min with 0.8% crystal violet. After removal of the excess of the dye, followed by rinsing and drying of the plate, crystal violet bound to the adherent cells was resolubilized in 96% ethanol and OD_570 nm_ was measured. All tested variants were carried out in eight replicates and the results were averaged.

### 3.6. Phylogenetic Analysis

The closest reference and representative genomes to that of O23S were identified by Mash/MinHash [53] as a part of PATRIC automatic annotation [26]. PATRIC global protein families were selected from these genomes to determine the phylogenetic location of the *Shewanella* sp. O23S genome. The protein sequences obtained from these families were aligned with MUSCLE [54], and the nucleotides for each of those sequences were mapped to the protein alignment. The combined set of amino acid and nucleotide alignments were concatenated into a data matrix, which was then analyzed by RAxML program with the rapid bootstrapping option to generate the support values in the tree [55].

### 3.7. Nucleotide Sequence Accession Numbers

The nucleotide sequences of *Shewanella* sp. O23S chromosome and extrachromosomal replicons pSheA-E have been annotated and deposited in the GenBank database (BioSample acc. no. SAMN10141545). The .gbk files may be found in the Supplementary Materials (Appendix B).

## 4. Conclusions

Members of *Shewanellaceae* are known for their involvement in the cycle of arsenic in the environment, most of them being related to mobilization of this element. The strain investigated in this work, *Shewanella* sp. O23S was isolated from microbial mats from the Zloty Stok mine (SW Poland), an environment strongly contaminated with arsenic. Based on the previous analyses it was found that O23S is one of the main driving agents of dissimilatory reduction in the mine population. The performed analyses help to further describe the role of not only *Shewanella* sp. O23S, but also other microorganisms sharing dissimilatory reduction properties in the environment, indicating that it may be more complex than previously thought. As it was shown, full-genome sequencing may help to assess the actual potential of the strain, also in relation to the benefits and risks of using it in bioremediation processes, including controlled release of arsenic from contaminated solids, soils, minerals, and industrial waste material. This can lead to the sequestration of not only toxic, but also potentially valuable metals. On the other hand, given the recent discoveries in the field of DARB-driven immobilization of heavy metals, this group of bacteria seems to have a significant impact on the circulation of metal species in the environment. Both physiological and genomic studies of O23S described in this work open up the possibility of testing other interesting abilities of this strain, which may further increase its importance—not only as the host of the pSheB plasmid being a unique vector of functional *ars* and *arr* genes, but also as a potential tool in bioremediation processes, no less important than the well-studied and industrially-applicable *Shewanella oneidensis*.

## 5. Patents

The pSheB plasmid and its functional derivatives are subject to Patent protection (US Patent no. 9328397B2).

## Figures and Tables

**Table 1:** General features of the *Shewanella* sp. O23S genome.

General Features	Chromosome	pSheA	pSheB	pSheC	pSheD	pSheE
size (bp)	5,098,757	111,800	81,605	8147	6658	14,805
GC content (%)	45.34	39.4	44.0	40.7	40.0	40.4
coding density (%)	85.40	86.3	89.4	87.7	90.7	89.0
number of genes	4654	157	91	12	11	23
number of tRNA genes	106	0	0	0	0	0
number of 16S-23S-5S rRNA gene clusters	10 (plus one additional gene for 5S rRNA)	0	0	0	0	0
phage regions	3	0	0	1	1	2

**Table 2:** Growth of strains with and without the *arr/ars* module on LB medium supplemented with As(III) under aerobic conditions. Heatmaps reflect the intensity of growth.

Strain	As(III) Concentration [mM]											
	0	1	2	3	4	5	6	7	8	9	10	11
*E. coli* F96401-1 (FOS 41C with *arr/ars*)												
*E. coli* F96401-1 (no fosmid)												
*E. coli* F96401-1 (fosmid without *arr/ars*)												
*Shewanella* sp. O23S												
Uninoculated medium (control)												

Color scale:
OD_600 nm_00.20.40.60.811.21.41.6

**Table 3:** Growth of strains with and without the arr/ars module on LB medium supplemented with As(V) under aerobic conditions. Heatmaps reflect the intensity of growth.

Strain	As(V) Concentration [mM]											
	0	25	50	100	150	200	250	300	350	400	450	500
*E. coli* F96401-1 (FOS 41C with *arr/ars*)												
*E. coli* F96401-1 (no fosmid)												
*E. coli* F96401-1 (fosmid without *arr/ars*)												
*Shewanella* sp. O23S												
Uninoculated medium (control)												

Color scale:
OD_600 nm_00.20.40.60.811.21.41.6

**Table 4:** Growth of *Shewanella* sp. O23S for 96 h at room temperature in MS medium (pH 7.5) without (**A**) or with (**B**) the addition of 10 mM Tris-HCl buffer. The media in respective variants were supplemented with As(III) (2.5 or 5 mM) or As(V) (2.5 or 5 mM) and 1 mM of Cu(II), Fe(III), Ni(II) or Zn(II). Media without arsenic were used as the control.

(**A**)		MS	MS + As(III) [mM]		MS + As(V) [mM]		(**B**)		MS	MS + As(III) [mM]		MS + As(V) [mM]	
		-	2.5	5	5	10			-	2.5	5	5	10
	-							-					
	Cu(II)							Cu(II)					
	Fe(III)							Fe(III)					
	Ni(II)							Ni(II)					
	Zn(II)							Zn(II)					

Color scale
OD_600 nm_0.00–0.050.05–0.100.10–0.15>0.15

**Table 5:** Putative antibiotic resistance genes of the O23S strain and MIC results.

No.	ORF no. (Chromosome)	Gene Name	Putative Product	Predicted Function	Result, MIC Value (mg/L)
1 2	2288 2289	*mex*	MexPQ-OpmE multidrug efflux pump	carbapenem, acridine dye, phenicol antibiotic, diaminopyrimidine, tetracycline, macrolide resistance	n–f., O23S is susceptible to: AMP—1.0; CFM—0.38; CTX—0.19; CRO—0.19; TE—0.25; E—0.75
3 4 5 6	2298 2517 3381 4409	*bcr*	Drug resistance transporter Bcr/CmlA	bicyclomycin, chloramphenicol and florfenicol resistance	n–f., O23S is susceptible to: C—0.75
7	2321	*mdtK/norM*	Multidrug efflux transporter MdtK/NorM (MATE family)	fluoroquinolone, tetraphenylphosphonium ion, deoxycholate, doxorubicin, trimethoprim, fosfomycin, ethidium bromide, benzalkonium, kanamycin and streptomycin resistance	n–f., O23S is susceptible to: CIP—0.016; MXF—0.004; TM—0.25; C—0.75
8	2377	*emrD*	EmrD (Multidrug resistance efflux pump)	aminoglycoside resistance	n–f., O23S is susceptible to: CN—0.75
9 10 11 12 13	880 2919 3979 3980 3132	*acrA* *acrB*	AcrA-B (Multidrug resistance efflux pump)	acriflavin, aminoglycoside, β-lactam, glycylcycline, macrolide resistance	n–f., O23S is susceptible to: AMP—1.0; CFM—0.38; CTX—0.19;CRO—0.19; E—0.75; CN—0.75
14 15	569 3371		Metallo-beta-lactamase superfamily protein	resistance to almost all clinically-available β-lactam antibiotics including carbapenems	n–f., O23S is susceptible to: AMP—1.0; CFM—0.38; CTX—0.19; CRO—0.19
16 17	3390 3391	*mexF* *mexE*	MexE/F-OprN	resistance to chloramphenicol and fluoroquinolones	n–f., O23S is susceptible to: CIP—0.016; MXF—0.004; C—0.75
18 19	3753 3752	*macB* *macA*	MacA/B (Macrolide-specific efflux system)	Macrolide resistance	n–f., O23S is susceptible to: E—0.75
20	4001	*mdtL*	MdtL. (Multidrug resistance efflux pump)	Chloramphenicol resistance	n–f., O23S is susceptible to: C—0.75
21 22 23	4552 4553 4554	*fus*	Fus	Fusaric acid resistance	-
24 25	847 2376		Beta-lactamase class C-like and penicillin binding proteins (PBPs)	Resistance to β-lactam antibiotics	n–f., O23S is susceptible to: AMP—1.0; CFM—0.38; CTX—0.19; CRO—0.19
26	3739		Class D beta-lactamase, OXA-48 family	Resistance to β-lactam antibiotics including carbapenems	n–f., O23S is susceptible to: AMP—1.0; CFM—0.38; CTX—0.19; —0.19

Abbreviations: n–f.—non-functional; AMP—ampicillin; C—chloramphenicol; CFM—cefixime; CRO—ceftriaxone; CTX—cefotaxime; E—erythrocin; TE—tetracycline.

## Supplementary Materials

Supplementary materials can be found at https://www.mdpi.com/1422-0067/20/5/1018/s1.

## Abbreviations

DARB	Dissimilatory arsenate reducing bacterium/bacteria
HGT	Horizontal Gene Transfer
hmr	Heavy metal resistance
MIC	Minimal Inhibitory Concentration
ORF	Open Reading Frame
