# Supplementary material for: Genomic Analysis of Shewanella sp. O23S—The Natural Host of the pSheB Plasmid Carrying Genes for Arsenic Resistance and Dissimilatory Reduction
Source: Int J Mol Sci. 2019 Feb 26;20(5):1018. doi: 10.3390/ijms20051018 (PMC6429310; doi:10.3390/ijms20051018)
Supplement: Supplementary file 1 [file ijms-20-01018-s001.zip › Appendix A.pdf]

Appendix A

Supplementary Files

1. Supplementary Tables

Table S1. CDSs of the plasmid pSheA.

ORF	Coding sequence position		Protein size (aa)	Possible function	ORF	Coding sequence position		Protein size (aa)	Possible function
	start codon	stop codon				start codon	stop codon		
pSheA 01	1	942	313	RepA family replication initiator protein	pSheA 80	51259	51909	216	hypothetical protein
pSheA 02	1643	2713	356	hypothetical protein	pSheA 81	51921	52343	140	hypothetical protein
pSheA 03	3453	4502	349	RepB family plasmid replication initiator protein	pSheA 82	52370	53590	406	hypothetical protein
pSheA 04	4733	4530	67	hypothetical protein	pSheA 83	53622	53804	60	hypothetical protein
pSheA 05	4828	5370	180	hypothetical protein	pSheA 84	53801	54073	90	hypothetical protein
pSheA 06	6452	5358	364	hypothetical protein	pSheA 85	54345	54554	69	hypothetical protein
pSheA 07	6781	6449	110	hypothetical protein	pSheA 86	54682	54861	59	hypothetical protein
pSheA 08	7085	7393	102	hypothetical protein	pSheA 87	54899	55186	95	hypothetical protein
pSheA 09	7493	7849	118	hypothetical protein	pSheA 88	55334	56476	380	GIY-YIG nuclease family protein
pSheA 10	7846	9516	556	DEAD/DEAH box helicase	pSheA 89	56633	58051	472	DNA cytosine methyltransferase
pSheA 11	9829	10419	196	hypothetical protein	pSheA 90	58350	59543	397	plasmid partition protein A
pSheA 12	10482	10979	165	hypothetical protein	pSheA 91	59543	60451	302	plasmid partition protein B
pSheA 13	11163	11552	129	hypothetical protein	pSheA 92	60521	60655	44	hypothetical protein
pSheA 14	13100	11622	492	hypothetical protein	pSheA 93	60856	62172	438	hypothetical protein
pSheA 15	13355	14038	227	hypothetical protein	pSheA 94	63081	62341	246	hypothetical protein
pSheA 16	14106	14855	249	hypothetical protein	pSheA 95	63559	63098	153	hypothetical protein
pSheA 17	14855	16405	516	hypothetical protein	pSheA 96	63711	64991	426	dbifunctional diguanylate cyclase/phosphodiesterase

pSheA 18	16418	17641	407	hypothetical protein	pSheA 97	66245	65058	395	hypothetical protein
pSheA 19	17657	18409	250	hypothetical protein	pSheA 98	66258	66419	53	hypothetical protein
pSheA 20	18418	20043	541	PulE	pSheA 99	66443	67357	304	ParA family protein
pSheA 21	20060	21178	372	hypothetical protein	pSheA 100	67376	68635	419	ParB-like partition protein
pSheA 22	21322	21744	140	prepilin-type N-terminal cleavage/methylation domain-containing protein, PulG	pSheA 101	68635	69069	144	hypothetical protein
pSheA 23	21834	22391	185	hypothetical protein	pSheA 102	69047	70516	489	hypothetical protein
pSheA 24	22504	25287	927	hypothetical protein	pSheA 103	70656	71948	430	hypothetical protein
pSheA 25	25344	26534	396	GlyGly-CTERM sorting domain-containing protein	pSheA 104	71950	72270	106	restriction endonuclease
pSheA 26	27128	28591	487	PcfJ-like protein	pSheA 105	72353	74167	604	DNA topoisomerase III
pSheA 27	28607	29197	196	hypothetical protein	pSheA 106	74179	74577	132	hypothetical protein
pSheA 28	29396	29719	107	hypothetical protein	pSheA 107	74683	75495	270	conjugative transfer protein TraP
pSheA 29	29890	30027	45	hypothetical protein	pSheA 108	75476	75802	108	hypothetical protein
pSheA 30	30152	29985	55	hypothetical protein	pSheA 109	75814	76530	238	hypothetical protein
pSheA 31	30790	30179	203	bacteriophage CI repressor	pSheA 110	76530	77072	180	lytic transglycosylase TraG
pSheA 32	31093	31350	85	hypothetical protein	pSheA 111	77036	77593	185	hypothetical protein
pSheA 33	31384	31956	190	hypothetical protein	pSheA 112	77593	79746	717	conjugative coupling factor TraD
pSheA 34	31969	32265	98	hypothetical protein	pSheA 113	79746	80444	232	hypothetical protein
pSheA 35	32267	32467	66	hypothetical protein	pSheA 114	80529	80867	112	hypothetical protein
pSheA 36	32603	32926	107	hypothetical protein	pSheA 115	80870	81154	94	hypothetical protein
pSheA 37	33345	33139	68	hypothetical protein	pSheA 116	81198	81581	127	hypothetical protein
pSheA 38	33596	33784	62	hypothetical protein	pSheA 117	81588	81956	122	hypothetical protein
pSheA 39	33869	34009	46	hypothetical protein	pSheA 118	81960	82598	212	membrane protein
pSheA 40	34077	34259	60	hypothetical protein	pSheA 119	82816	85122	768	ATP-binding protein
pSheA 41	34303	34791	162	hypothetical protein	pSheA 120	85257	86024	255	hypothetical protein

pSheA 42	34829	35020	63	hypothetical protein	pSheA 121	86024	87469	481	integrating conjugative element protein
pSheA 43	35150	35455	101	hypothetical protein	pSheA 122	87466	87819	117	hypothetical protein
pSheA 44	35442	35705	87	hypothetical protein	pSheA 123	87821	90844	1007	conjugative transfer ATPase
pSheA 45	35717	36007	96	hypothetical protein	pSheA 124	91212	90895	105	hypothetical protein
pSheA 46	36017	36298	93	hypothetical protein	pSheA 125	91358	91801	147	hypothetical protein
pSheA 47	36319	36795	158	hypothetical protein	pSheA 126	91798	92904	368	integrating conjugative element protein
pSheA 48	36815	37078	87	hypothetical protein	pSheA 127	92908	94245	445	integrating conjugative element protein
pSheA 49	37109	37336	75	hypothetical protein	pSheA 128	94254	94592	112	hypothetical protein
pSheA 50	37352	37888	178	hypothetical protein	pSheA 129	94623	96377	584	hypothetical protein
pSheA 51	37875	38072	65	hypothetical protein	pSheA 130	96686	96943	85	hypothetical protein
pSheA 52	38102	38347	81	antirestriction protein ArdR	pSheA 131	97494	97769	91	hypothetical protein
pSheA 53	38359	38721	120	hypothetical protein	pSheA 132	97857	98360	167	hypothetical protein
pSheA 54	38826	39038	70	hypothetical protein	pSheA 133	98544	99197	217	resolvase
pSheA 55	39032	39277	81	hypothetical protein	pSheA 134	99424	99645	73	hypothetical protein
pSheA 56	39297	39665	122	hypothetical protein	pSheA 135	99797	100318	173	hypothetical protein
pSheA 57	39816	40100	94	hypothetical protein	pSheA 136	100447	101832	461	hypothetical protein
pSheA 58	40129	40668	179	hypothetical protein	pSheA 137	102035	102619	194	methyltransferase type11
pSheA 59	40693	40914	73	hypothetical protein	pSheA 138	102701	103081	126	N-acetyltransferase
pSheA 60	40936	41151	71	hypothetical protein	pSheA 139	103174	103413	79	hypothetical protein
pSheA 61	41179	41439	86	antirestriction protein ArdR	pSheA 140	103570	103824	84	hypothetical protein
pSheA 62	41442	41789	115	hypothetical protein	pSheA 141	103943	104227	94	hypothetical protein
pSheA 63	41805	42050	81	hypothetical protein	pSheA 142	104459	104286	57	hypothetical protein
pSheA 64	42074	42361	95	hypothetical protein	pSheA 143	104810	105196	128	hypothetical protein
pSheA 65	42443	42838	131	hypothetical protein	pSheA 144	105330	105548	72	hypothetical protein

pSheA 66	42880	43107	75	hypothetical protein	pSheA 145	105693	106007	104	hypothetical protein
pSheA 67	43141	43392	83	hypothetical protein	pSheA 146	106133	106282	49	hypothetical protein
pSheA 68	43431	43736	101	hypothetical protein	pSheA 147	106432	106869	145	hypothetical protein
pSheA 69	43726	44136	136	hypothetical protein	pSheA 148	107063	107569	168	hypothetical protein
pSheA 70	44231	44587	118	hypothetical protein	pSheA 149	107724	108077	117	hypothetical protein
pSheA 71	44600	44839	79	hypothetical protein	pSheA 150	108282	108650	122	hypothetical protein
pSheA 72	44911	45375	154	hypothetical protein	pSheA 151	108920	109201	93	transcriptional regulator
pSheA 73	45599	46459	286	hypothetical protein	pSheA 152	109213	109545	110	hypothetical protein
pSheA 74	46645	48507	620	hypothetical protein	pSheA 153	109564	109806	80	hypothetical protein
pSheA 75	48550	48852	100	hypothetical protein	pSheA 154	109817	110254	145	hypothetical protein
pSheA 76	48958	49287	109	hypothetical protein	pSheA 155	110351	110620	89	hypothetical protein
pSheA 77	49586	49771	61	hypothetical protein	pSheA 156	110645	110800	51	hypothetical protein
pSheA 78	49853	50974	373	DNA sulfur modification protein	pSheA 157	111626	111336	96	hypothetical protein
pSheA 79	51027	51221	64	hypothetical protein					

The ORFs forming the pSheA plasmid backbone structure modules and their putative protein products are color-coded: replication – beige, conjugal transfer – blue, partitioning system – green, multimer resolution system – grey.

Table S2. CDSs of the plasmid pSheB.

ORF	Coding sequence position		Protein size (aa)	Possible function	ORF	Coding sequence position		Protein size (aa)	Possible function
	start codon	stop codon				start codon	stop codon		
pSheB 01	1	435	144	DNA polymerase V	pSheB 47	40303	39929	124	hypothetical protein TraA
pSheB 02	423	1676	417	DNA polymerase V subunit UmuC	pSheB 48	40555	40367	62	TraY domain-containing protein
pSheB 03	1775	1990	71	hypothetical protein	pSheB 49	40914	40657	85	hypothetical protein
pSheB 04	2096	2746	216	hypothetical protein	pSheB 50	41034	41216	60	hypothetical protein
pSheB 05	2787	3005	72	hypothetical protein	pSheB 51	41227	41376	49	hypothetical protein

pSheB 06	3025	3429	134	hypothetical protein
pSheB 07	3426	4157	243	endonuclease I
pSheB 08	4172	4405	77	hypothetical protein
pSheB 09	4548	4402	48	hypothetical protein
pSheB 10	4798	6672	624	ParB domain protein nuclease
pSheB 11	6797	7252	151	hypothetical protein
pSheB 12	7254	7742	162	hypothetical protein
pSheB 13	7773	7994	73	hypothetical protein
pSheB 14	8007	8120	37	hypothetical protein
pSheB 15	8365	8237	42	hypothetical protein
pSheB 16	8359	8703	114	hypothetical protein
pSheB 17	8785	9291	168	hypothetical protein
pSheB 18	9293	9694	133	hypothetical protein
pSheB 19	9892	10137	81	HicA protein
pSheB 20	10137	10472	111	HicB family protein
pSheB 21	10776	10648	42	hypothetical protein
pSheB 22	10770	11114	114	hypothetical protein
pSheB 23	11077	12405	442	hypothetical plasmid protein
pSheB 24	13003	13314	103	hypothetical protein
pSheB 25	13439	13311	42	hypothetical protein
pSheB 26	13872	14231	119	hypothetical protein
pSheB 27	20206	14252	1984	conjugative transfer relaxase/helicase TraI
pSheB 28	20444	20265	59	thyroid hormone receptor interactor 11-like protein
pSheB 29	22584	20458	708	type IV conjugative transfer system coupling protein TraD

pSheB 52	41658	42590	310	hypothetical protein
pSheB 53	43512	43793	93	toxin-antitoxin system antidote Rhh family
pSheB 54	43799	44326	175	N-acetyltransferase
pSheB 55	44914	44768	48	hypothetical protein
pSheB 56	45164	46363	399	chromosome partitioning protein ParA
pSheB 57	46363	47460	365	chromosome partitioning protein ParB
pSheB 58	47633	47755	40	hypothetical protein
pSheB 59	47791	51651	1286	hypothetical protein
pSheB 60	51741	52466	241	hypothetical protein
pSheB 61	52599	52976	125	hypothetical protein
pSheB 62	53014	53898	294	hypothetical protein
pSheB 63	54008	54976	322	hypothetical protein
pSheB 64	55266	55739	157	hypothetical protein
pSheB 65	56576	55947	209	resolvase
pSheB 66	56699	57019	106	transposase
pSheB 67	57098	57478	126	membrane protein YnfA
pSheB 68	57887	58195	102	ArsR family transcriptional regulator
pSheB 69	58256	58498	80	Redox-active disulfide protein 2
pSheB 70	58517	59050	177	sulfite exporter TauE/SafE family protein
pSheB 71	59043	59741	232	Cytochrome c biogenesis protein, transmembrane region
pSheB 72	59786	60910	374	efflux RND transporter periplasmic adaptor subunit
pSheB 73	60907	63981	1024	efflux RND transporter permease subunit
pSheB 74	64533	64099	144	arsenate reductase ArsC
pSheB 75	65904	64621	427	arsenical efflux pump membrane protein ArsB

pSheB 30	23219	22584	211	hypothetical protein	pSheB 76	67741	65969	590	arsenical pump-driving ATPase ArsA
pSheB 31	26070	23257	937	conjugative transfer protein TraG	pSheB 77	68138	67776	120	arsenical resistance operon transcriptional repressor ArsD
pSheB 32	27461	26073	462	conjugative transfer protein TraH	pSheB 78	68511	71075	854	respiratory arsenate reductase ArrA
pSheB 33	27634	27458	58	hypothetical protein	pSheB 79	71087	71791	234	respiratory arsentate reductase ArrB
pSheB 34	28085	27645	146	type-F conjugative transfer system pilin assembly thiol-disulfide isomerase TrbB	pSheB 80	73292	71862	476	glutathione synthase
pSheB 35	28974	28099	291	type-F conjugative transfer system pilin assembly protein TraF	pSheB 81	74500	73448	350	hypothetical protein
pSheB 36	30782	28971	603	type-F conjugative transfer system mating-pair stabilization protein TraN	pSheB 82	74725	75012	95	ArsR family transcriptional regulator
pSheB 37	31519	30779	246	type-F conjugative transfer system pilin assembly protein TrbC	pSheB 83	75111	75527	138	arsenate reductase ArsC
pSheB 38	32549	31542	335	conjugative transfer protein TraU	pSheB 84	75520	75867	115	ArsR family transcriptional regulator
pSheB 39	33240	32536	234	type-F conjugative transfer system protein TraW	pSheB 85	75946	76458	170	arsenate reductase ArsC
pSheB 40	33608	33237	123	conjugative transfer protein TrbI	pSheB 86	76551	77549	332	hypothetical protein
pSheB 41	36198	33610	862	type IV secretion system protein TraC	pSheB 87	77561	77797	78	thioredoxin family protein
pSheB 42	36657	36202	151	type IV conjugative transfer system protein TraV	pSheB 88	77805	78296	163	protein phosphatase
pSheB 43	38204	36678	508	conjugative transfer protein TraB	pSheB 89	78353	79363	336	glyceraldehyde-3-phosphate dehydrogenase
pSheB 44	39016	38201	271	type-F conjugative transfer system secretin TraK	pSheB 90	79369	80607	412	major facilitator superfamily transporter
pSheB 45	39582	39016	188	type IV conjugative transfer system protein TraE	pSheB 91	80738	81013	91	hypothetical protein
pSheB 46	39925	39623	100	type IV conjugative transfer system protein TraL					

The ORFs forming the pSheB plasmid backbone structure modules and their putative protein products are color-coded: replication – beige, conjugal transfer – blue, partitioning system – green, multimer resolution system – grey, addiction module – red. The *ars* and *arr* modules components are colored in yellow. The putive ParB protein, which may also play a role in plasmid replication is colored in gold.

Table S3. ORFs located within phage MuSsp_O23S

ORF	Coding sequence position		Protein size (aa)	Possible function
	start codon	stop codon		

MuSsp_O23S 1	905	183	240	Bifunctional HTH-domain containing protein/aminotransferase with peptidase S24-like domain; transcriptional regulator protein (CI repressor)
MuSsp_O23S 2	1071	1319	82	HTH-XRE regulatory protein; (Mu-like phage Ner protein)
MuSsp_O23S 3	1312	3471	719	Mu-like phage transposase A
MuSsp_O23S 4	3542	4261	239	Mu-like phage transposase B
MuSsp_O23S 5	4317	4883	188	Hypothetical protein
MuSsp_O23S 6	4870	5364	164	XRE family transcriptional regulator
MuSsp_O23S 7	5364	5582	72	Mu Kil-like protein
MuSsp_O23S 8	5584	6219	211	Hypothetical protein, DUF3164; sulfate transporter
MuSsp_O23S 9	6221	6547	108	Hypothetical protein
MuSsp_O23S 10	6639	6860	73	Hypothetical protein
MuSsp_O23S 11	6854	7474	206	Mu GemA-like; host gene modulation protein, DUF1018
MuSsp_O23S 12	7461	7922	153	Mor transcription activator domain protein (Mu-like phage late transcription activator C)
MuSsp_O23S 13	8429	9337	302	Clp protease
MuSsp_O23S 14	9381	9938	185	Hypothetical protein
MuSsp_O23S 15	9967	10401	144	Hypothetical protein
MuSsp_O23S 16	10408	10433	102	E18 Hypothetical protein
MuSsp_O23S 17	10784	11344	186	Mu-like phage lysozyme (endolysin)
MuSsp_O23S 18	11341	11910	189	Mu-like phage LysB, phage lysis regulatory protein
MuSsp_O23S 19	11995	12219	74	C4 DksA/TraR-type zinc finger protein
MuSsp_O23S 20	12216	12554	112	Hypothetical protein containing a coiled-coil domain
MuSsp_O23S 21	12554	12850	98	Mu Gp 26-like protein; Ribonuclease R winged-helix domain protein
MuSsp_O23S 22	12853	13125	90	Hypothetical protein
MuSsp_O23S 23	13115	13684	189	Mu Gp27 (GpD)-like protein; small terminase subunit
MuSsp_O23S 24	13684	15324	546	Mu Gp28 (GpE)-like protein; large terminase subunit
MuSsp_O23S 25	15324	16925	533	Mu-like phage portal protein
MuSsp_O23S 26	16918	18243	441	Mu Gp30 (GpF)-like protein; head assembly protein
MuSsp_O23S 27	18253	18714	153	Mu Gp31 (GpG)-like protein, capsid morphogenesis protein
MuSsp_O23S 28	19551	18697	284	HrpJ-like domain containing protein
MuSsp_O23S 29	20281	20529	103	XRE family transcriptional regulator
MuSsp_O23S 30	20567	21523	318	Reverse transcriptase (RNA-dependent DNA polymerase)

MuSsp_O23S 31	21644	21970	110	Hypothetical protein
MuSsp_O23S 32	22050	23207	385	Mu-like phage protease I
MuSsp_O23S 33	23207	24127	306	Major head subunit (GpT-like)
MuSsp_O23S 34	24210	24722	170	Mu Gp35-like protein; Rho termination factor, N-terminal domain
MuSsp_O23S 35	24725	25165	146	Mu Gp36 (Gp J)-like protein
MuSsp_O23S 36	25165	25608	147	Virion structural protein
MuSsp_O23S 37	25637	26383	248	Hypothetical protein
MuSsp_O23S 38	26503	30429	1308	Tail length tape measure protein
MuSsp_O23S 39	30426	30791	121	Hypothetical protein
MuSsp_O23S 40	30804	33278	824	Hypothetical protein
MuSsp_O23S 41	33290	34249	319	Hypothetical protein
MuSsp_O23S 42	34242	34544	100	Hypothetical protein
MuSsp_O23S 43	34541	36328	595	Hypothetical protein
MuSsp_O23S 44	36325	36546	73	Hypothetical protein
MuSsp_O23S 45	36692	37453	253	DNA adenine methyltransferase

Table S4. ORFs located within pSheC.

ORF	Coding sequence position		Protein size (aa)	Possible function	% identity with proteins encoded in pSheD and pSheE	Homologues
	start codon	stop codon				
pSheC 01	66	320	84	Hypothetical protein	pSheE 14 100%	
pSheC 02	501	283	72	Transcriptional regulator	pSheE 15 100%	
pSheC 03	729	505	74	Hypothetical protein	pSheE 16 100%	
pSheC 04	1318	1115	67	Hypothetical protein	pSheE 17 100%	
pSheC 05	1454	3598	714	RstA, replication-associated protein	pSheE 18 100%	YP_004286230 Vibrio phage CTXphi (50.4%)
pSheC 06	3658	3972	104	RstB, integration (lysogeny)-associated protein	pSheE 19 100%	YP_004286231 Vibrio phage CTXphi (49.5%)
pSheC 07	3985	4191	68	Minor virion protein, pVII	pSheD 07 42%, pSheE 20 100%	Vibrio virus Vf33
pSheC 08	4201	4410	69	Major virion protein, pVIII	pSheD 09 50%, pSheE 21 100%	NP_932299 Ralstonia phage p12J 39%

pSheC 09	4485	5942	485	Attachment protein, Receptor-binding protein, pIII	pSheD 09 78%, pSheE 22 100%	NP_047369 Vibrio phage fs2, 21%
pSheC 10	5951	6298	115	Minor virion protein, pVI	pSheD 10 78%, pSheE 24 100%	NP_932301 Ralstonia phage p12J 27%
pSheC 11	6298	7605	435	Assembly protein, Zonular occludens toxin, pI	pSheD 11 58%, pSheE 25 100%	NP_932302 Ralstonia phage p12J 37.3%
pSheC 12	7618	7875	85	Hypothetical protein	pSheE 26 100%	

Table S5. ORFs located within pSheD.

ORF	Coding sequence position		Protein size (aa)	Possible function	% identity with proteins encoded in pSheC and pSheE	Homologues
	start codon	stop codon				
pSheD 01	245	505	86	Hypothetical protein	pSheE 01 100%	
pSheD 02	495	686	142	Hypothetical protein	pSheE 02 100%	
pSheD 03	1138	902	78	Mobilization protein BmgB	pSheE 04 100%	
pSheD 04	1168	2328	386	Replication protein (endonuclease), pII	pSheE 05 100%	NP_047361 of Enterobacteria phage If1 64.1%, IKE, fl
pSheD 05	2029	2328	99	Replication-associated protein, pX	pSheE 06 100%	
pSheD 06	2342	2665	107	ssDNA binding protein (replication), pV	pSheE 07 100%	IKE, fl
pSheD 07	2678	2881	67	Minor virion protein, pVII	pSheC 07 42%, pSheE 08 100%	
pSheD 08	2899	3117	72	Major virion protein, pVIII	pSheC 08 50%, pSheE 09 100%	
pSheD 09	3187	4668	493	Receptor binding protein, pIII	pSheC 09 78%, pSheE 10 100%	
pSheD 10	4676	5023	115	Minor virion protein, pVI	pSheC 10 78%, pSheE 12 100%	
pSheD 11	5023	6333	436	Assembly protein, Zonular occludens toxin, pI	pSheC 11 58%, pSheE 13 100%	

a -the nomenclature of Ff (M13, f1 and fd) phage proteins is used

Table S6. Biofilm production indicator (OD_{570}/OD_{600} ratio) values in LB medium and LB supplemented with 5 mM of As(III) or As(V).

	Time [hours]		
Medium	24	48	72
LB	0.769	0.597	0.293
LB+As(III)	1.284	0.381	0.306
LB+As(V)	1.516	0.344	0.218

2. Supplementary Figures

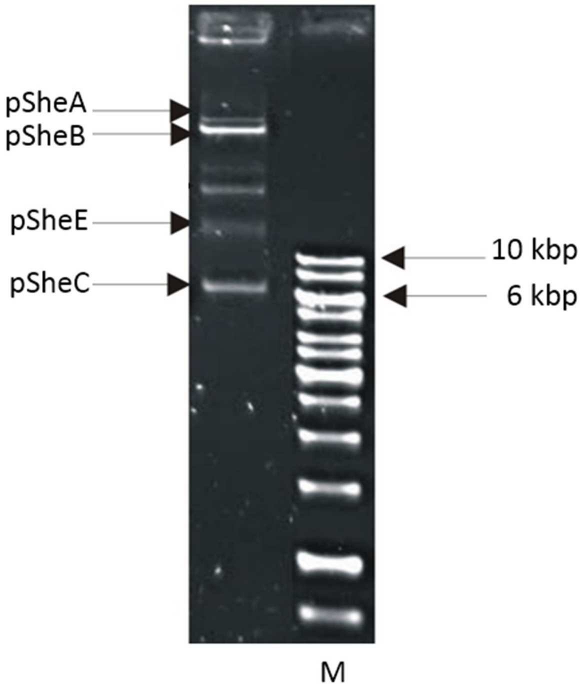

Figure S1. Agarose gel electrophoresis of the DNA of extrachromosomal replicons of *Shewanella* sp. O23S. Plasmid pSheD is not visible. Multimer forms of the plasmids pSheC-D can be seen

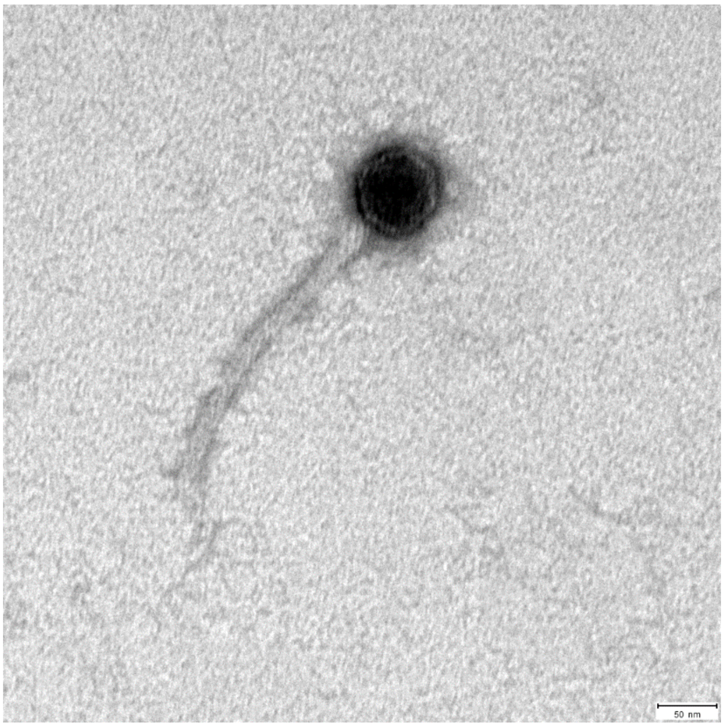

Figure S2. Transmission electron microscopy image of the phage MuSsp1_O23S of *Shewanella* sp. O23S.

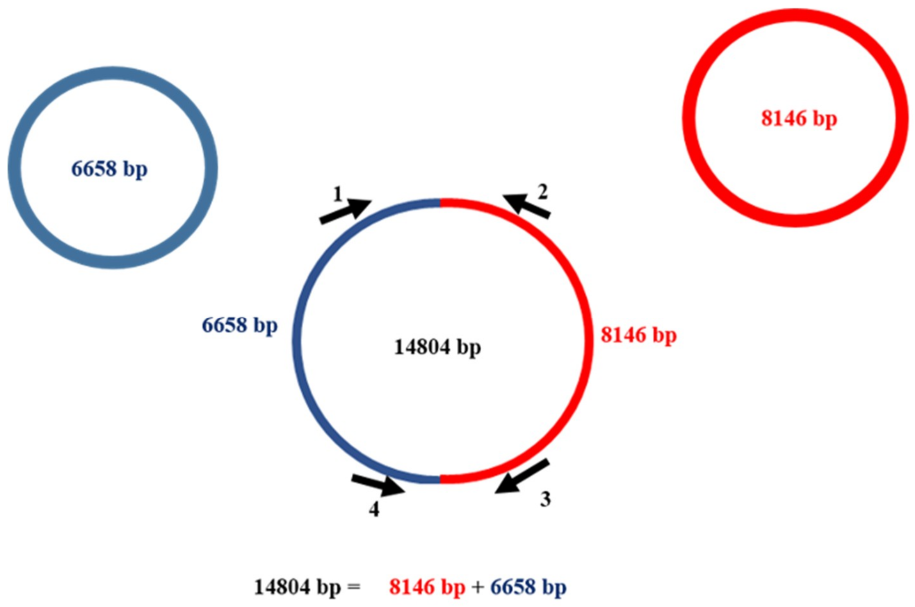

Figure S3. Plasmid-like phage pSheE (cointegrate of pSheC and pSheD) identification strategy. Arrows with numbers indicate primers, used in pairs: 1-2, 3-4, 1-4 and 2-3.

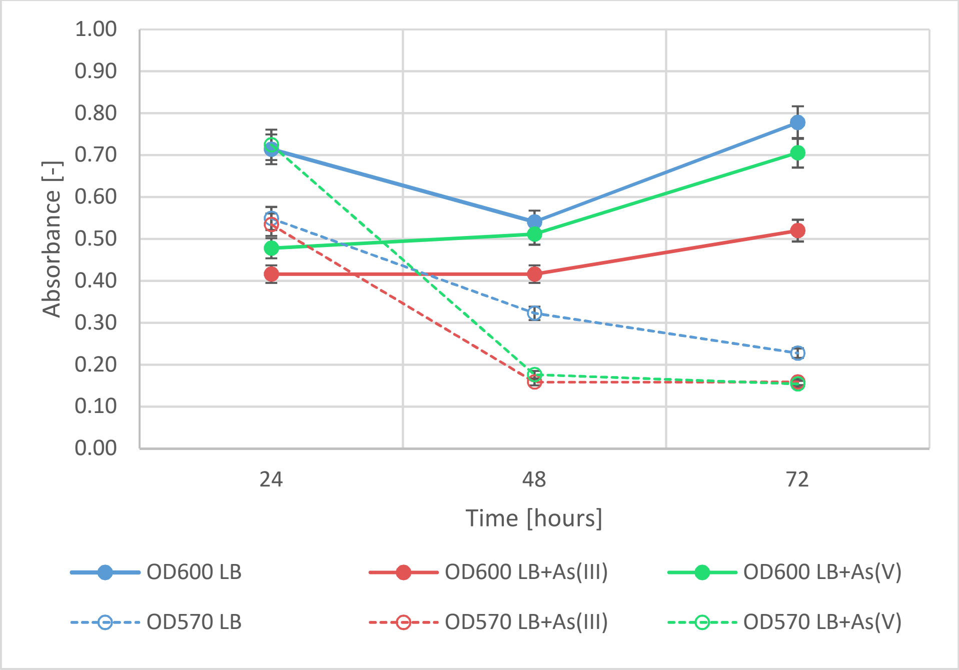

Figure S4. Changes in optical density of the O23S culture ($OD_{600\ nm}$) in LB medium (with or without arsenic) and in the number of adherent cells stained with crystal violet ($OD_{570\ nm}$).

Negative Control	L-Arabinose	N-Acetyl-D-Glucosamine	D-Saccharic Acid	Succinic Acid	Succinic Acid	L-Aspartic Acid	L-Proline	D-Alanine	D-Trehalose	D-Mannose	Dulcitol
D-Serine	D-Sorbitol	Glycerol	L-Fucose	D-Glucuronic Acid	D-Gluconic Acid	D,L-α-Glycerol-Phosphate	D-Xylose	L-Lactic Acid	Formic Acid	D-Mannitol	L-Glutamic Acid
D-Glucose-6-Phosphate	D-Galactonic Acid-γ-Lactone	D,L-Malic Acid	D-Ribose	Tween 20	L-Rhamnose	D-Fructose	Acetic Acid	α-D-Glucose	Maltose	D-Melibiose	Thymidine
L-Asparagine	D-Aspartic Acid	D-Glucosaminic Acid	1,2-Propanediol	Tween 40	α-Keto-Glutaric Acid	α-Keto-Butyric Acid	α-Methyl-D-Galactoside	α-D-Lactose	Lactulose	Sucrose	Uridine
L-Glutamine	m-Tartaric Acid	D-Glucose-1-Phosphate	D-Fructose-6-Phosphate	Tween 80	α-Hydroxy Glutaric Acid- -Lactone	α-Hydroxy Butyric Acid	β-Methyl-D-Glucoside	Adonitol	Maltotriose	2-Deoxy Adenosine	Adenosine
Glycyl-L-Aspartic Acid	Citric Acid	m-Inositol	D-Threonine	Fumaric Acid	Bromo Succinic Acid	Propionic Acid	Mucic Acid	Glycolic Acid	Glyoxylic Acid	D-Cellobiose	Inosine
Glycyl-L-Glutamic Acid	Tricarballylic Acid	L-Serine	L-Threonine	L-Alanine	L-Alanyl-Glycine	Acetoacetic Acid	N-Acetyl-β-D-Mannosamine	Mono Methyl Succinate	Methyl Pyruvate	D-Malic Acid	L-Malic Acid
Glycyl-L-Proline	p-Hydroxy Phenyl Acetic Acid	m-Hydroxy Phenyl Acetic Acid	Tyramine	D-Psicose	L-Lyxose	Glucuronamide	Pyruvic Acid	L-Galactonic Acid-γ-Lactone	D-Galacturonic Acid	Phenylethylamine	2-Aminoethanol

Figure S5. Biolog™ PM1 MicroPlate carbon sources analysis for *Shewanella* sp. O23S. Color intensity indicates substrate preference.

Negative Control	Chondroitin Sulfate C	α-Cyclodextrin	β-Cyclodextrin	γ-Cyclodextrin	Dextrin	Gelatin	Glycogen	Inulin	Laminarin	Mannan	Pectin
N-Acetyl-D-Galactosamine	N-Acetyl-Neuraminic Acid	β-D-Allose	Amygdalin	D-Arabinose	D-Arabitol	L-Arabitol	Arbutin	2-Deoxy-D-Ribose	i-Erythritol	D-Fucose	3-0-β-D-Galacto-pyranosyl-D-Arabinose
Gentiobiose	L-Glucose	Lactitol	D-Melezitose	Maltitol	α-Methyl-D-Glucoside	β-Methyl-D-Galactoside	3-Methyl Glucose	β-Methyl-D-Glucuronic Acid	α-Methyl-D-Mannoside	β-Methyl-D-Xyloside	Palatinose
Raffinose	Salicin	Sedoheptulosan	L-Sorbose	Stachyose	D-Tagatose	Turanose	Xylitol	N-Acetyl-D-Glucosaminitol	γ-Amino Butyric Acid	δ-Amino Valeric Acid	Butyric Acid
Capric Acid	Caproic Acid	Citraconic Acid	Citramalic Acid	D-Glucosamine	2-Hydroxy Benzoic Acid	4-Hydroxy Benzoic Acid	β-Hydroxy Butyric Acid	γ-Hydroxy Butyric Acid	α-Keto-Valeric Acid	Itaconic Acid	5-Keto-D-Gluconic Acid
D-Lactic Acid Methyl Ester	Malonic Acid	Melibionic Acid	Oxalic Acid	Oxalomalic Acid	Quinic Acid	D-Ribono-1,4-Lactone	Sebacic Acid	Sorbic Acid	Succinamic Acid	D-Tartaric Acid	L-Tartaric Acid
Acetamide	L-Alaninamide	N-Acetyl-L-Glutamic Acid	L-Arginine	Glycine	L-Histidine	L-Homoserine	Hydroxy-L-Proline	L-Isoleucine	L-Leucine	L-Lysine	L-Methionine
L-Ornithine	L-Phenylalanine	L-Pyroglutamic Acid	L-Valine	D,L-Carnitine	Sec-Butylamine	D.L-Octopamine	Putrescine	Dihydroxy Acetone	2,3-Butanediol	2,3-Butanone	3-Hydroxy 2-Butanone

Figure S6. Biolog™ PM2A MicroPlate carbon sources analysis for *Shewanella* sp. O23S. Color intensity indicates substrate preference.

Negative Control	Ammonia	Nitrite	Nitrate	Urea	Biuret	L-Alanine	L-Arginine	L-Asparagine	L-Aspartic Acid	L-Cysteine	L-Glutamic Acid
L-Glutamine	Glycine	L-Histidine	L-Isoleucine	L-Leucine	L-Lysine	L-Methionine	L-Phenylalanine	L-Proline	L-Serine	L-Threonine	L-Tryptophan
L-Tyrosine	L-Valine	D-Alanine	D-Asparagine	D-Aspartic Acid	D-Glutamic Acid	D-Lysine	D-Serine	D-Valine	L-Citrulline	L-Homoserine	L-Ornithine
N-Acetyl-L-Glutamic Acid	N-Phthaloyl-L-Glutamic Acid	L-Pyroglutamic Acid	Hydroxylamine	Methylamine	N-Amylamine	N-Butylamine	Ethylamine	Ethanolamine	Ethylenediamine	Putrescine	Agmatine
Histamine	β-Phenylethyl-amine	Tyramine	Acetamide	Formamide	Glucuronamide	D,L-Lactamide	D-Glucosamine	D-Galactosamine	D-Mannosamine	N-Acetyl-D-Glucosamine	N-Acetyl-D-Galactosamine
N-Acetyl-D-Mannosamine	Adenine	Adenosine	Cytidine	Cytosine	Guanine	Guanosine	Thymine	Thymidine	Uracil	Uridine	Inosine
Xanthine	Xanthosine	Uric Acid	Alloxan	Allantoin	Parabanic Acid	D,L-α-Amino-N-Butyric Acid	γ-Amino-N-Butyric Acid	δ-Amino-N-Caproic Acid	D,L-α-Amino-Caprylic Acid	δ-Amino-N-Valeric Acid	α-Amino-N-Valeric Acid
Ala-Asp	Ala-Gln	Ala-Glu	Ala-Gly	Ala-His	Ala-Leu	Ala-Thr	Gly-Asn	Gly-Gln	Gly-Glu	Gly-Met	Met-Ala

Figure S7. Biolog™ PM3B Microplate nitrogen sources analysis for *Shewanella* sp. O23S. Color intensity indicates substrate preference.

Negative Control	Phosphate	Pyrophosphate	Trimeta-phosphate	Tripoly-phosphate	Triethyl Phosphate	Hypophosphite	Adenosine-2'-monophosphate	Adenosine-3'-monophosphate	Adenosine-5'-monophosphate	Adenosine-2',3'-cyclic monophosphate	Adenosine-3',5'-cyclic monophosphate
Thiophosphate	Dithiophosphate	D,L-α-Glycerol Phosphate	β-Glycerol Phosphate	Carbamyl Phosphate	D-2-Phospho-Glyceric Acid	D-3-Phospho-Glyceric Acid	Guanosine-2'-monophosphate	Guanosine-3'-monophosphate	Guanosine-5'-monophosphate	Guanosine-2',3'-cyclic monophosphate	Guanosine-3',5'-cyclic monophosphate
Phosphoenol Pyruvate	Phospho-Glycolic Acid	D-Glucose-1-Phosphate	D-Glucose-6-Phosphate	2-Deoxy-D-Glucose 6-Phosphate	D-Glucosamine-6-Phosphate	6-Phospho-Gluconic Acid	Cytidine- 2'-monophosphate	Cytidine- 3'-monophosphate	Cytidine- 5'-monophosphate	Cytidine-2',3'-cyclic monophosphate	Cytidine-3',5'-cyclic monophosphate
D-Mannose-1-Phosphate	D-Mannose-6-Phosphate	Cysteamine-S-Phosphate	Phospho-L-Arginine	O-Phospho-D-Serine	O-Phospho-L-Serine	O-Phospho-L-Threonine	Uridine- 2'-monophosphate	Uridine- 3'-monophosphate	Uridine- 5'-monophosphate	Uridine- 2',3'-cyclic monophosphate	Uridine- 3',5'-cyclic monophosphate
O-Phospho-D-Tyrosine	O-Phospho-L-Tyrosine	Phosphocreatine	Phosphoryl Choline	O-Phosphoryl-Ethanolamine	Phosphono Acetic Acid	2-Aminoethyl Phosphonic Acid	Methylene Diphosphonic Acid	Thymidine-3'-monophosphate	Thymidine-5'-monophosphate	Inositol Hexaphosphate	Thymidine 3',5'- cyclic monophosphate
Negative Control	Sulfate	Thiosulfate	Tetrathionate	Thiophosphate	Dithiophosphate	L-Cysteine	D-Cysteine	L-Cysteinyl-Glycine	L-Cysteic Acid	Cysteamine	L-Cysteine Sulfinic Acid
N-Acetyl-L-Cysteine	S-Methyl-L-Cysteine	Cystathionine	Lanthionine	Glutathione	D,L-Ethionine	L-Methionine	D-Methionine	Glycyl-L-Methionine	N-Acetyl-D,L-Methionine	L-Methionine Sulfoxide	L-Methionine Sulfone
L-Djenkolic Acid	Thiourea	1-Thio-β-D-Glucose	D,L-Lipoamide	Taurocholic Acid	Taurine	Hypotaurine	p-Amino Benzene Sulfonic Acid	Butane Sulfonic Acid	2-Hydroxyethane Sulfonic Acid	Methane Sulfonic Acid	Tetramethylene Sulfone

Figure S8. Biolog™ PM4A MicroPlate phosphorus (green) and sulfur (orange) sources analysis for *Shewanella* sp. O23S.
